# Differing effects of size and lifestyle on bone structure in mammals

**DOI:** 10.1186/s12915-021-01016-1

**Published:** 2021-04-29

**Authors:** Eli Amson, Faysal Bibi

**Affiliations:** 1 Museum für Naturkunde, Leibniz Institute for Evolution and Biodiversity Science, Invalidenstraße 43, 10115 Berlin, Germany; 2 Staatliches Museum für Naturkunde Stuttgart, Rosenstein 1, 70191 Stuttgart, Germany

**Keywords:** Bone structure, Constructional morphology, Lifestyle convergence, Mammals, Size effect

## Abstract

**Background:**

Mammals are a highly diverse group, with body mass ranging from 2 g to 170 t, and encompassing species with terrestrial, aquatic, aerial, and subterranean lifestyles. The skeleton is involved in most aspects of vertebrate life history, but while previous macroevolutionary analyses have shown that structural, phylogenetic, and functional factors influence the gross morphology of skeletal elements, their inner structure has received comparatively little attention. Here we analysed bone structure of the humerus and mid-lumbar vertebrae across mammals and their correlations with different lifestyles and body size.

**Results:**

We acquired bone structure parameters in appendicular and axial elements (humerus and mid-lumbar vertebra) from 190 species across therian mammals (placentals + marsupials). Our sample captures all transitions to aerial, fully aquatic, and subterranean lifestyles in extant therian clades. We found that mammalian bone structure is highly disparate and we show that the investigated vertebral structure parameters mostly correlate with body size, but not lifestyle, while the opposite is true for humeral parameters. The latter also show a high degree of convergence among the clades that have acquired specialised (non-terrestrial) lifestyles.

**Conclusions:**

In light of phylogenetic, size, and functional factors, the distribution of each investigated structural parameter reveals patterns explaining the construction of appendicular and axial skeletal elements in mammalian species spanning most of the extant diversity of the clade in terms of body size and lifestyle. These patterns should be further investigated with analyses focused on specific lifestyle transitions that would ideally include key fossils.

## Background

The skeleton in vertebrates is involved in many important biological roles, such as supporting body weight, locomotion, and feeding [1–3]. Numerous investigations of the gross morphology of skeletal elements (i.e. their outer shape) have revealed complex relationships with ecology or life history (e.g. [4, 5]). In contrast, the macroevolutionary analyses of bone structure, namely the distribution of bone tissue within a skeletal element (e.g. [6–10]), are comparatively scarce, creating a major impediment to our understanding of vertebrate evolution. Bone structure is known for its high degree of plasticity [11]. Indeed, it was suggested as early as the late nineteenth century that bone structure can adapt to the mechanical loads applied to skeletal elements throughout life [11] (Wolff’s law, or *bone functional adaptation*). In the context of comparative analyses, however, it is impossible to disentangle the effect of phenotypic plasticity from evolutionary acquisitions. Furthermore, it is likely that the reaction norm (potential plastic response) of bone structure is under selective pressure and can itself evolve [12]. In such analyses, a particular phenotype is rather argued to be the result of structural, phylogenetic, functional, and environmental factors [13–15]. This was for instance demonstrated for the gross morphology of the appendicular and axial skeleton, with comparative analyses of the forelimb [16] in musteloids and the mandible [17] in ungulates. The extent to which lifestyle, phylogenetic heritage, body size, and other factors influence bone structure at a broad macroevolutionary scale is poorly understood.

This study investigates bone structure and its correlates across therian mammals (placentals + marsupials). They range from ca. 2 g (Etruscan shrew) to up to 170 t (blue whale), and occupy a diverse array of environments, from the depths of the ocean to aerial heights. The evolutionary history of mammals is marked by a series of diversification events, resulting in the acquisition of various specialised lifestyles as early as the Jurassic in early mammaliaforms [18]. However, it is assumed that each large mammalian clade, the marsupials and placentals in particular, stems from small-sized, terrestrial (or scansorial), insectivorous, or omnivorous ancestors that diversified independently after the Cretaceous Terrestrial Revolution [18]. The subsequent evolution of each mammal clade saw transitions to highly specialised lifestyles strongly departing from those reconstructed for these Cretaceous forms. Arguably the most extreme are the aerial, fully aquatic, and subterranean lifestyles which were each acquired convergently on several independent occasions in marsupials and placentals [19–21].

We investigate the correlation between bone structure and the species’ size and lifestyle in a phylogenetically informed context. We focused our analyses on lumbar vertebrae and the humerus, these elements being generally conserved (and not vestigial) across mammals, and having been previously associated with locomotor adaptations reflected in their gross morphology [16, 22, 23]. Since mammals (and other amniotes) share broadly similar ontogenetic trajectories (in contrast to anurans for instance [5]) and bony tissue types [24], a low disparity in bone structure across the clade should be interpreted as indicative of preponderant structural constraints. Strong phylogenetic constraints (or inertia) should in turn be reflected by a strong phylogenetic signal (but see [25]). On the other hand, a dominant influence of functional factors is expected to result in clear correlation between trait distributions and lifestyle. Similarly, the influence of body size can be investigated by examining the correlation of traits with a size proxy. The diversity of mammalian bone structure is here captured by quantifying humeral and vertebral traits for highly specialised taxa (aerial, fully aquatic, and subterranean), their terrestrial sister groups (TSG), and more distantly related terrestrial species. We use these data to test whether differential patterns in the structure of the appendicular and axial skeleton can be correlated with these specialised lifestyles, taking into account species body size and phylogenetic relationships.

## Results

### Lifestyle transitions in mammals

**Fig. 1:**
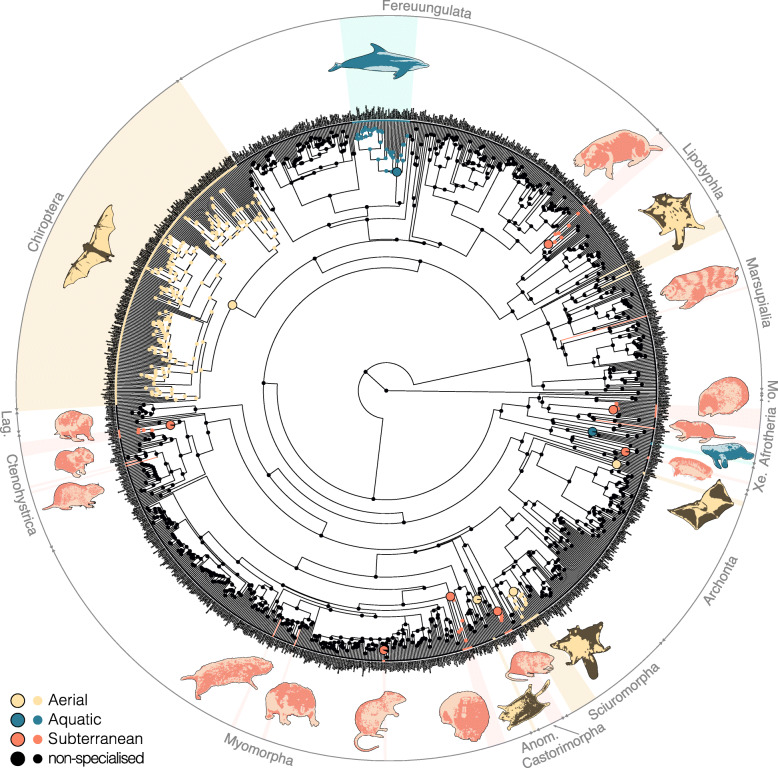
Lifestyle transitions among mammals. Colours correspond to the specialised lifestyles, i.e. aerial, aquatic, and subterranean; black corresponds to ‘non-specialised’ lifestyles. States at the nodes are reconstructed with stochastic character mapping. Lifestyle transitions were emphasised with larger nodes for clades and coloured branches for single taxa. Each silhouette represents an independent acquisition of one of the three specialised lifestyles. Abbreviations: Anom., Anomaluromorpha; Lag., Lagomorpha; Mo., Monotremata; Xe, Xenarthra

As indicated by stochastic character mapping, all reconstructed transitions to a specialised lifestyle on the tree are supported by a high posterior probability. These include no reversions (Fig. 1). From an ancestrally non-specialised lifestyle, transition events led to two aquatic convergences, cetaceans, and sirenians; 13 subterranean convergences, eight in rodents, two in afrotheres, one in xenarthrans, and one in marsupials; and seven aerial convergences, three in marsupials, two in rodents, and one each in archontans, and chiropterans. The sister groups of each of the marsupial clades that have acquired gliding are arboreal and are hence excluded from the subsequent analyses (see ‘Methods’). The latter will consequently recognise only one acquisition of the aerial lifestyle in marsupials, and five aerial convergences in total (see also Additional file 1, Figure S1).

### Size effect and lifestyle signal

**Fig. 2:**
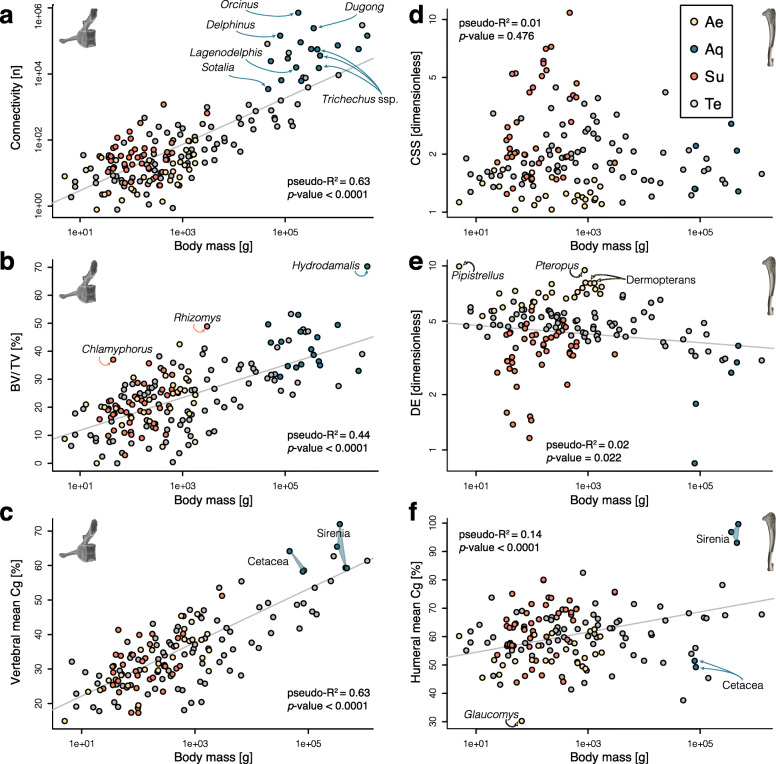
Size effect accounting for phylogeny on the studied bone structure parameters across lifestyles. Vertebral (**a**–**c**) and humeral (**d**–**f**) parameters are plotted against body mass. Colours and grey indicate aerial (Ae), aquatic (Aq), subterranean (Su), and terrestrial (Te) lifestyles

**Fig. 3:**
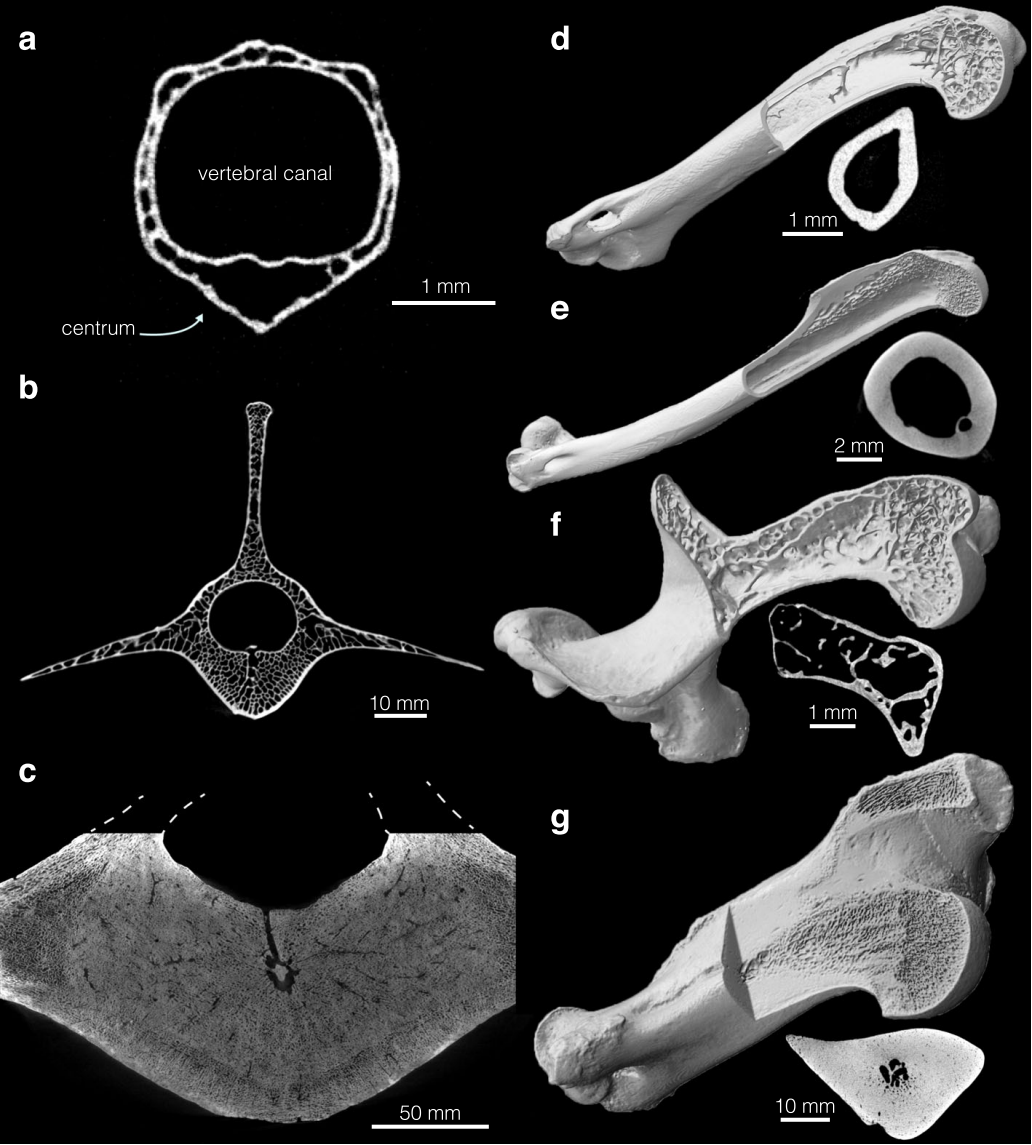
Disparity of vertebral and humeral structure among mammals. Transverse cross section of the lumbar vertebrae at centrum’s mid-length (dorsal towards the top) for **a** Hose’s pygmy flying squirrel (*Petaurillus hosei*; NHMUK ZD 1900.7.29.26), **b** fallow deer (*Dama dama*; ZMB_Mam_94752), **c** Steller’s sea cow (*Hydrodamalis gigas*; MNHN_AC_1919–48). Humerus 3D rendering (not to scale) and midshaft cross section for **d** the long-eared gymnure (*Hylomys megalotis*; NHMUK ZD 1999.47), **e** Sunda colugo (*Galeopterus variegatus*; ZMB_Mam_69096), **f** southern marsupial mole (*Notoryctes typhlops*; ZMB_Mam_35694), (**g**) dugong (*Dugong dugon* ZMB_Mam_69340)

**Fig. 4:**
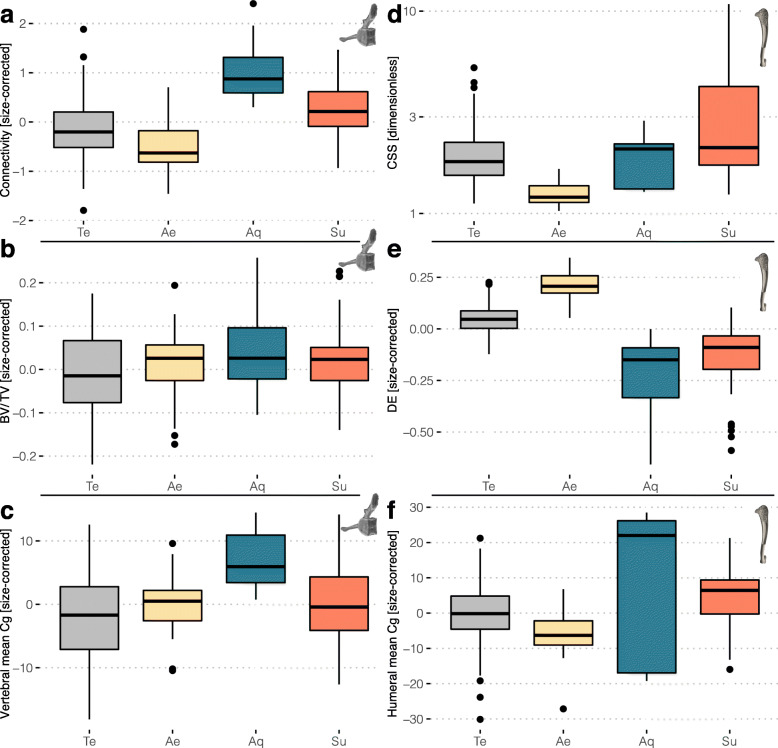
Differences among lifestyles (or the lack thereof) in the studied bone structure parameters. Boxplots (centre line, median; box limits, upper and lower quartiles; whiskers, 1.5 times interquartile range; points, outliers) depicting the distribution of **a**–**c** vertebral parameters and **d**–**f** humeral parameters. Abbreviations: Ae, aerial; Aq, aquatic; Te, terrestrial; Su, subterranean

Body size affects preponderantly the investigated vertebral structure parameters among mammals. Mean vertebral global compactness (the ratio of cross-sectional area occupied by bone to total cross-sectional area, Cg [26]) and trabecular architecture within the vertebral body (centrum)—the Connectivity (approximates the number of trabeculae) and BV/TV (fraction of bone contained in the volume of interest [VOI] over total VOI volume) show a strong positive correlation with size, even when lifestyle is accounted for, as indicated by phylogenetically informed ANCOVAs (pANCOVAs, *p* values < 0.0001; pseudo *R*^2^ = 0.44–0.68) and phylogenetically informed regression of the parameter against body mass (pANOVAs, *p* values < 0.0001, pseudo *R*^2^ = 0.44–0.63; Fig. 2a–c; Additional file 3C; see also regressions against a specimen-specific size proxy in Additional file 3D). Connectivity Density (Conn.D) correlates negatively with body size, but the associated coefficient denotes positive allometry (Additional file 1, Figure S2). Some small-sized taxa display surprisingly few (if any) trabeculae in the core of their centrum (low Connectivity; Figs. 2a, 3a), which therefore presents a low bone fraction (BV/TV; Fig. 2b). The latter conclusion can probably be extended to the whole vertebra, as their mean vertebral Cg is also particularly low (Fig. 2c). As they increase in size, mammals of all lifestyles show a similar increase of vertebral Cg, Connectivity, and BV/TV (Figs. 2 and 3b), which is reflecting positive allometry for these three dimensionless parameters (slopes all significantly greater than 0, Additional file 3C-D; the absence of slope would denote isometry). Accordingly, none of the vertebral traits display a lifestyle signal (Fig. 4a–c) with three exceptions (also found when accounting for size effect and when the terrestrial sample is pruned to match the size of the specialised taxa): the aquatic taxa depart from the terrestrial ones in having a higher mean vertebral Cg (pANCOVAs *p* values = 0.015); aquatic taxa also differ from other lifestyles with higher Connectivity (*p* values < 0.023), and the subterranean taxa differ from the terrestrial and aerial taxa in featuring a higher Connectivity (*p* values < 0.008). Conn.D’s distribution pattern broadly mirrors that of Connectivity (Additional file 1, Figure S2; value range 0.4–208 trabeculae/mm^3^; see also descriptive statistics in Additional file 3B) and will hence not be examined further.

Conversely, there is no preponderant size effect on the investigated humeral parameters: the mean humeral Cg, cross-sectional shape (CSS), and diaphysis elongation (DE) (pANOVAs parameter ~ size, all pseudo *R*^2^ < 0.14; Fig. 2d-f; Additional file 3C). Only DE seems to consistently scale (with weak negative allometry) across lifestyles (pANCOVA, *p* value < 0.005; Fig. 2e). The correlation between mean humeral Cg and body mass (pANCOVA, *p* value = 0.002) is mostly driven by the high values of the large-sized sirenians (Fig. 2f). A clear lifestyle signal is found in the humeral structure, including when the size effect is accounted for and when the terrestrial sample is pruned to match the size of the specialised taxa (Fig. 2d–f; Fig. 4d–f; Additional file 3C). DE, in particular, differs quite consistently among lifestyles (Fig. 2e; Fig. 4e). Aerial taxa (Fig. 3e) differ from subterranean (Fig. 3f) and terrestrial taxa (Fig. 3d) in featuring a diaphysis that is more elongate (higher DE; pANCOVA *p* values < 0.001) and also more circular in cross section (lower CSS; *p* values < 0.002). Furthermore, subterranean taxa differ from terrestrial taxa in featuring a lower DE (*p* value < 0.001) and higher CSS (*p* value < 0.020). Aerial and subterranean taxa were also found as having respectively higher and lower DE values than the aquatic ones (*p* values < 0.005), but the lack of size overlap between the groups prevents a strict exclusion of the size effect.

### Convergence analysis

**Fig. 5:**
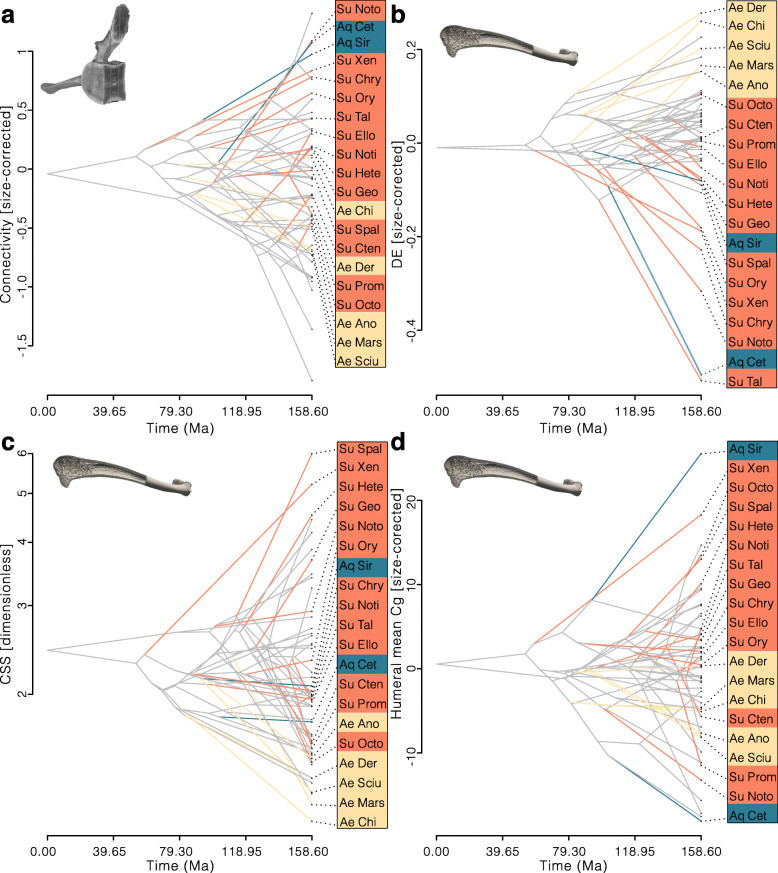
Trait convergence among mammalian lifestyles. Phenograms depicting reconstructed evolution of the vertebral centrum Connectivity (**a**) and humeral diaphysis elongation (**b**), midshaft cross-sectional shape (**c**), and mean global compactness (**d**). Note the number of branches leading to the specialised clades (coloured) evolving in the same direction for converging traits (e.g. in **a** twelve out of 13 subterranean clades increased their Connectivity when compared to their direct ancestor). Clade abbreviations: see Additional file 3A. Lifestyle abbreviations: Ae, aerial; Aq, aquatic; Te, terrestrial; Su, subterranean

Examination of the strength and direction of the convergence in the studied parameters’ evolution allows to better understand the modalities by which each specialised lifestyle was acquired among mammals. For the subterranean lifestyle, acquired by 13 clades, the Connectivity (corrected for size effect; vertebral centrum VOI) do show overall convergence (C1 = 0.50, *p* value < 0.001) with twelve clades increasing their mean value compared to the reconstructed value of their respective ancestral nodes (five exceeding the corresponding 95% confidence intervals [95CIs]; Fig. 5a). DE (humerus) on the other hand does not show strong overall convergence across subterranean taxa (C1 = 0. 24, *p* value = 0.91; using size-corrected values), but eleven clades decreased their values (six exceeding the 95CIs; Fig. 5b). No strong overall convergence is found in CSS (humerus) of subterranean taxa either (C1 = 0.28, *p* value = 0.72), and seven clades show an increase of their values (four exceeding the 95CI; Fig. 5c). The distribution of all traits, including the other, non-converging ones, can be found in Additional file 3B.

Among aerial taxa, overall convergence is the clearest for CSS (C1 = 0.71, *p* value < 0.001), with all five clades decreasing their values compared to their respective ancestral nodes (three exceeding the 95CIs). DE also shows overall convergence (C1 = 0.59, *p* value < 0.01), with all clades showing an increase of their values (all exceeding the 95CIs). Humeral mean Cg does also converge among aerial clades (C1 = 0.52, *p* value < 0.01), with four clades showing a decrease (two exceeding the 95CIs). While Connectivity does not show clear evidence of convergent evolution (C1 = 0.40; *p* value = 0.059), four out of five clades feature a decrease of their values (one exceeding the 95CI).

For aquatic clades, only BV/TV (size-corrected; vertebral centrum VOI) shows a strong overall convergence (C1 = 0.94; *p* value < 0.01), but only cetaceans increased their values when compared to their ancestral node (not exceeding the 95 CI). One parameter is actually diverging between the two aquatic clades: the humeral mean Cg increased in sirenians while it decreased in cetaceans (exceeding the 95CIs for the former; Fig. 5d). The absence of overall convergences for the vertebral mean Cg might be due in part to the size correction we used for this parameter, which weakens the signal (uncorrected values fall beyond most of other lifestyle’s values). For this parameter both clades show an increase of their values (only cetaceans exceed the corresponding 95CI). The same is true for the Connectivity (C1 = 0.90; *p* value = 0.053), for which both clades feature an increase of their values (cetaceans exceed the 95CIs).

### Phylogenetic signal

A significant phylogenetic signal was found for all but two traits in terrestrial taxa when all species were analysed independently (lambdas = 0.47–0.85; *p* values < 0.008; Additional file 3E). However, when the members of the TSG are aggregated to sample the mammalian tree more uniformly, none of the traits were showing a significant phylogenetic signal except for the Connectivity and DE (lambdas = 0.74–0.79; *p* values < 0.042).

## Discussion

### Body size and bone structure

The influence of body size on vertebral structure is most compelling when examining the smallest species. The mid-lumbar vertebra of small mammals is often delicate: the overall compactness (mean vertebral Cg) can be as low as 15% and no or few trabeculae are observed the middle of the centrum (Figs. 2 and 3). This is in contrast to the structure commonly observed in larger species: a centrum entirely filled with spongy bone, and an overall compactness of ca. 30–50%. A more robust construction of vertebrae with increasing size is therefore the rule in mammals, as all vertebral traits of our dataset scale with positive allometry (Fig. 2; including the Connectivity Density, Additional file 1, Figure S2). A similar conclusion was reached for the dorsal vertebrae of squamates [27]. Several vertebral gross morphological traits were shown to scale with allometry in terrestrial mammals [22, 28, 29]. These have been correlated to an increased stiffness of the vertebral column accompanying larger size. Both gross morphology and structure are hence in accordance with an area to volume scaling [30], which predicts that stresses will increase with body mass because the strength of the vertebral column only scales with the cross-sectional area. That biomechanical interpretation should be tempered regarding the mean vertebral Cg, however, because the vertebral canal seems to scale with negative allometry (see, e.g. Fig. 3a, b). While this scaling was not assessed by our analysis, this seems to be confirmed by the fact that spinal cord weight scales with negative allometry in mammals [31]. However, this is not minoring the positive allometry we recovered for the trabecular traits, because the latter were acquired in a volume of interest placed within the centrum (body of the vertebra). In mammals, the centrum is expected to mainly withstand axial compression during bending of the vertebral column [32]. Using a finite element analysis of the human centrum loaded in compression, it was shown that relative loads shared by the cortex and trabecular bone depend on the distance from the cranial and caudal ends of the centrum [33]: the greatest fraction of load taken by the cortex (up to 54% of the total load) was found in the middle of centrum. Our data suggest that under a certain body size, loads could be low enough to be mostly (if not entirely) withstood by non-trabecular tissues.

### Lifestyle signal

#### Aquatic lifestyle and vertebral structure

The aquatic lifestyle is the class for which we found the clearest association with specific vertebral structures (also humeral structure, see below). This could be ascribed to the drastically different nature of the aquatic environment, which relieves the vertebral column from the loads associated with a quadrupedal stance and terrestrial locomotion (notably due to Archimedes’ principle). Indeed, other constraints are associated with aquatic locomotion, and two major bone structure adaptations are assumed to relate to buoyancy and trim control during swimming [34]. Bone mass increase (BMI; classified as non-pathological pachyostosis and/or osteosclerosis) has been reported in sirenians, and interpreted in light of their shallow diving habits [35]. Conversely, qualitative assessments of cetacean vertebral structure have reported bone mass decrease, notably due to thinner cortices [36]. However, previous quantification of the 2D bone structure of the centrum did not reveal considerable differences of bone fraction compared to their terrestrial relatives [37, 38], which was recently confirmed with a 3D microarchitecture analysis of the cetacean thoracic, lumbar, and caudal vertebrae [39]. This is also what is indicated by the ANCOVA we performed on the bone fraction within the centrum (BV/TV, Fig. 2b; Additional file 3C). Although this trait was found as converging in our analysis, values did not conspicuously increase in either clade when compared to their respective reconstructed ancestral values (Additional file 4). Our analysis more clearly revealed that the overall vertebral bone fraction (mean vertebral Cg) is high in both sirenians and cetaceans (Fig. 2c). Because both parameters are affected by positive allometry, and because sirenians and cetaceans are among the largest mammals included in the dataset, it is difficult to safely associate high mean vertebral Cg with an aquatic lifestyle (as taking into account size as covariate or using size correction will be least accurate for extreme values). A large body size is itself a trait associated with the fully aquatic lifestyle. Based on our analysis, there is nevertheless no reason to acknowledge overall vertebral BMI in sirenians and not in cetaceans. The convergence analysis even showed that the latter more sharply increase from the corresponding reconstructed ancestral value. Both sirenians and cetacean might hence share similar functional constraints upon overall vertebral bone fraction. The conclusion has to be tempered by the extreme case of Steller’s sea cow (*Hydrodamalis gigas*; Fig. 3c), its centrum’s bone fraction exceeding 70%. Such a value, unparalleled in extant mammals, confirms that with the recent extinction of this species we lost a truly exceptional component of mammalian diversity [40]. Vertebral BMI was also qualitatively reported in one desmostylian (extinct order of marine mammals [41]). Regarding the similar patterns observed in mean vertebral Cg and BV/TV, it should be noted that these parameters are partly redundant because part of centrum VOI belongs to the region sampled for vertebral Cg.

We also found that the number of trabeculae (Connectivity) is greater than expected for their size in sirenian and cetaceans (Figs. 2a, 4a, and 5a). Accordingly, average mean trabecular spacing (measured for a subset of species, see ‘Methods’) was found to be lower than in terrestrial and aerial taxa, while the opposite was true for mean trabecular thickness (Additional files 2B and 4a-b). Dumont et al. [38] interpreted a similar observation (therein based on other trabecular parameters, the relative trabecular length and trabecular proliferation) in pelagic species as potentially related to protecting the vertebra from accumulating fatigue microfractures. This type of damage is assumed to occur during the locomotion of these species, which entails high frequency cycles of tension and compression exerted on the vertebral centra. Our results concur quite well with this interpretation, with manatees and coastal cetaceans featuring relatively less trabeculae in their centrum (accounting for the size effect on Connectivity) than the more active pelagic cetaceans and dugong [42] (Fig. 2a). On the other hand, increased Connectivity in aquatic taxa, which are all of large size, could be due to the fact that the trabeculae of larger species were found to be often pierced with the canals of secondary osteons (‘osteonal tunnelling’ [43]). However, qualitative inspections of our scans confirmed that trabeculae were relatively small and tightly packed in aquatic taxa; osteonal tunnelling was not extensively observed.

#### Subterranean lifestyle and vertebral structure

The other vertebral parameter that was found in our analysis to correlate with the investigated lifestyles is the Connectivity (which approximates the number of trabeculae). Many subterranean species are characterised by a greater number of trabeculae than expected for their size (Figs. 2a, 4a, and 5a; Additional file 3C). This can be interpreted as indicative of greater axial compressive loads on the vertebral column [44]. Such greater loads could be expected to be associated to subterranean lifestyle: during the propulsion phase of digging, the vertebral column transmits the soil reaction force (through the hind limbs that anchor the body to the substrate [45]). The degree of anisotropy, which reflects whether the trabecular are preferentially aligned in one or more directions (measured for a subset of species, see ‘Methods’) was found to be particularly high in subterranean taxa. This could be consistent with greater axial compressive loads, as the trabeculae’s main direction is anteroposterior (Additional file 5c-d). However, such direction is found in virtually all taxa, and an effect of size is hard to exclude. Most subterranean taxa are small, which entails fewer trabeculae and which can in turn be associated with a greater degree of anisotropy. The strength and stiffness of trabecular bone being mostly due to its bone fraction [46], BV/TV (and to a lesser extent the mean vertebral Cg as well), should similarly be expected to be greater in subterranean species, which was not clearly featured by our data. Nevertheless, some subterranean taxa we have sampled, such as the pink fairy armadillo (*Chlamyphorus truncatus*), were marked by outstandingly high BV/TV values (Additional file 4). For the other subterranean species, a BV/TV value that is not particularly high but associated with a greater number of trabeculae (higher Connectivity) should entail for the latter to be thinner (which is not conspicuous in the subset of species for whom it was measured; Additional file 5a). In the context of bone loss, it has been shown that the number of trabeculae rather than their thickness is a major contributor of the strength of trabecular bone [47, 48]. Our results hence suggest that some subterranean clades might have developed a vertebral column structure capable of withstanding relatively greater loads without increasing its overall bone content, except for some cases for which additional strengthening might be required.

#### Lifestyles and humeral cross-sectional shape

Sharply differing from the vertebral bone structure, humeral structure correlates with each investigated lifestyle rather than body size. The cross-sectional shape (CSS; how elliptical/circular is the diaphysis in cross section) differentiates well the aerial and subterranean clades (Figs. 2d, 4a, and 5c). Each of the five aerial clades convergently acquired a more circular diaphysis in cross section (lower CSS values; Fig. 3e). Bats have the most circular diaphysis. The opposite trend is associated with the subterranean lifestyle, with in particular the spalacids (blind mole-rats and allies), heterocephalids (naked mole-rat and allies), geomyidae (pocket gophers), and the pink fairy armadillo that acquired a highly elliptical humeral diaphysis in cross section (Fig. 3f). Our assessment of CSS therefore illustrates quite well the spectrum of constraints acting on the humeral diaphysis, ranging from round cross sections (with relatively thin cortex, see below) that maximise resistance to torsional stresses in volant taxa [49] to the more elliptical cross sections that better withstand uniaxial bending loads associated with digging [50]. Strongly elliptical sections can also be associated with pronounced bone processes, and hence affect muscle conformation. At midshaft, a protruding deltoid tubercle, for instance, may provide better mechanical advantage for the humeral rotators in fossorial taxa (e.g. spinodeltoid in xenarthrans [51]). The low CSS in the non-volant aerial mammals, i.e. the gliding clades, could be reflective of the need to resist torsional loads as in bats and/or to a multidirectional bending environment, which is assumed in the case of the similarly rounder cross sections of non-aerial, arboreal mammals [52]. The low level of convergence we found for CSS among subterranean clades likely emphasises the disparity of fossorial behaviours, which for instance include scratch- and humeral rotation digging [53]. The talpids, who exemplify the latter digging style, were not characterised by elliptical cross sections (CSS values are not particularly high and not increased relative to the ancestral value; Fig. 5c). However, their humeral structure is clearly specialised, which is for instance demonstrated by their more elliptical medullary cavity [54]. It is nevertheless noteworthy that the humerus of other mammalian fossors has been shown to feature relatively high values of cross-sectional parameters such as the second moment of area or the polar section modulus [10, 55].

#### Lifestyles and humeral diaphysis elongation

The humeral diaphysis elongation (DE) also differentiates well the aerial and subterranean clades (Figs. 2e, 4a, and 5b), with elongate bones for the former (Fig. 3e) and stouter bones for the latter (Fig. 3f). Aquatic taxa also have a stout humerus (falling in the range of subterranean clades), but that is mostly expressed in cetaceans, and convergence is hence weak in their case. As with the cross-sectional shape, the aerial clades are featuring the strongest convergence, with each clade displaying a more elongate diaphysis when compared to that of their respective ancestral node. This is in accordance with the fact that these converging clades were also associated with a more regularly tubular structure of their humeral diaphysis [56]. While this trait scales with negative allometry for all other taxa (being the only humeral trait that shows a rather clear correlation with size), aerial species are clearly differing in showing positive allometry (Fig. 2e). This follows the expectation that wing surface should increase with positive allometry to be able to support increasing mass [57]. We do however find that the smallest sampled bat (*Pipistrellus pipistrellus*) outstands drastically from this scaling in featuring an exceptionally elongate humerus. Colugos (dermopterans) are recognised as differing from other gliding mammals notably regarding the construction of their patagium, somewhat approaching the chiropteran condition [58]. It is hence not surprising that both clades feature the most elongate diaphysis of our dataset (high DE values; Fig. 5b). The average DE value is actually lower in bats than in colugos. This likely emphasises that the handwing—trivially present in gliding mammals—is a key chiropteran autapomorphy [59]. Several gross anatomical features, among which longer and more gracile limbs, were found to have convergently evolved—though ‘incompletely’—among gliding mammals [60]. Only a weak convergence was found for DE among subterranean clades (C1 = 0. 24), but it is noteworthy that all clades but one (*Spalacopus cyanus*, the coruro) decreased their values when compared to their respective ancestral nodes (Fig. 5b). This might be indicative of the disparate subterranean adaptations (as they are associated with various degrees of fossoriality and digging styles) that are nevertheless sharing one basic feature, a relatively high bending strength for the humerus. It is rather clear that a strong negative allometry affects this trait in subterranean taxa, differing from the positive or weak negative scaling relationships found for the other lifestyles (see above; Fig. 2e).

Differing in that regard from the other investigated humeral traits, DE correlates quite clearly with body size. This trait naturally relates to humeral length, which has been shown to scale with weak negative allometry in mammals and tetrapods in general [61] (body mass ~ humeral length^2.9^; scaling coefficient with our dataset pruned to terrestrial species: humeral length^2.8^). This is consistent with the weak, negative allometry we found for DE. Our data however suggest that this allometric effect (DE ~ body mass^−0.023^; dataset pruned to terrestrial species: body mass^−0.035^; isometric coefficient for this correlation = 0) is minor when compared to the differences that can be imputed to lifestyle, as aerial and subterranean taxa feature conspicuously elongate or stouter bones, respectively (absolute value of coefficients > 0.11; Additional file 3C).

#### Lifestyles and humeral global compactness

The mean humeral global compactness (Cg), which as measured here reflects the bone fraction of the diaphysis, in turn most clearly discriminates those bone structure adaptations that relate to buoyancy and trim control [34]. The dichotomy between the sirenian bone mass increase (osteosclerosis; Fig. 3g) and cetacean osteoporosis-like pattern is compelling, and logically recovered as strongly diverging between the two clades in our analyses (Figs. 2f and 5d). The examination of this trait also allows to tackle the question of lightweightness among mammals [62]. The ANCOVA we performed suggests that the specialised lifestyles are not associated with differences in humeral Cg. However, the convergence analysis interestingly showed that this trait does converge among aerial clades, especially for bats, anomalurids (scaly tailed squirrels), and gliding sciurids (squirrels) that have acquired low Cg values (Fig. 5d). There is no major difference in the bone tissue density in mammals, which has been demonstrated in particular for the humerus of bats and various rodents [62]. One can therefore truly recognise a tendency for bats, scaly tailed squirrels, and gliding squirrels to have acquired a lightweight humerus. Because the humeral diaphysis of all the aerial species we sampled was basically tubular and of consistent Cg along the diaphysis, one can assimilate the mean humeral Cg to the relative cortical area (or thickness, also equivalent to the parameter K [63]) The average value for these species is 53%, which falls in the upper range of the relative cortical area of volant birds [64, 65].

### Differential microanatomical patterns

All bone structure parameters we investigated were found to be correlated with body size and/or lifestyle. Controlling for the potential effects of lifestyle and phylogeny, size is clearly correlated with all investigated vertebral traits. With the notable exception of the aquatic lifestyle, analyses of covariance and quantification of convergence suggest that lifestyle exerts little influence on these traits. As with several gross morphological traits of the vertebral column in (semi-)aquatic mammals [22, 23], we found that the vertebral structure of aquatic species differs from that of their terrestrial relatives (beyond what can be expected from a size effect alone). However, the other specialised lifestyles we investigated cannot be clearly associated with a particular vertebral phenotype. The opposite is true for the humeral traits we investigated, which vary according to lifestyle but show little or no influence of size. Although we did not assess intraspecific variation, each lifestyle acquisition is represented by several species, which can therefore be viewed as incorporating this potential variability to some extent.

Our results reveal a previously unsuspected diversity of inner vertebral anatomy in therian mammals, with, for example, an overall bone fraction ranging from ~ 15 to 72% (Fig. 3; Additional file 3B). High disparity in humeral structure was also observed, with the bone fraction ranging from ~ 30 to over 99% for the humeral diaphysis. These are among the most extreme values ever quantified for amniotes [66, 67], which lends support to the conclusion that ontogenetic constraints are not acting strongly on the bone structure of mammals (or only secondarily, such as the early growth of marsupials that might have made the acquisition of an aquatic lifestyle less likely). Furthermore, we found only weak phylogenetic signal in the investigated traits when mammals were sampled uniformly. While that does not strictly entail the absence of phylogenetic constraints [25], it indicates that phylogeny explains very little of the observed trait distributions.

The compartments making up the humerus and vertebrae are dependent on different ossification patterns, which makes strict inter-element comparisons difficult. Furthermore, the definition of all acquired parameters but one (global compactness, Cg) differs according to the sampled skeletal element, so it might be specious to conclude that size in vertebrae and lifestyle in the humerus should be viewed as the main factors determining bone structure in the mammalian postcranium. Given the investigated parameters, which we selected to capture the mechanical constraints acting on the skeletal elements with restrictions stemming from the overall size and epiphyseal fusion of some taxa (see ‘Methods’), it is nevertheless the conclusion supported by our analysis.

## Conclusions

The bone structure of two representative skeletal elements of the appendicular and axial skeleton—the mid-lumbar vertebra and the humerus—was found to be highly disparate across therian mammals. The distribution of the investigated vertebral traits was predominantly explained by body size but not their lifestyle, while the opposite was true for humeral traits. But this general pattern has to be nuanced for the fully aquatic and subterranean lifestyles, for which the values of some of the investigated structural parameters do depart from the generalised, terrestrial condition. In our extant sample, subterranean, aerial, and fully aquatic lifestyles were convergently acquired 13, five (or seven), and two times, respectively. These convergence events are detectable in the phylogenetic distribution of humeral bone structure. These conclusions should be corroborated with a more exhaustive assessment of therian evolution, which could be undertaken by sampling extinct representatives of these highly specialised lifestyles, such as the gliding eomyids [68] or glirids [69].

## Methods

Previous examinations of several levels of organisation of bone structure, be it histology [70, 71], microanatomy (e.g. trabecular architecture [43, 50, 72–75]), or gross morphology [22, 23], documented various patterns of correlation with species’ phylogenetic relationships, lifestyle, and body size. This work attempts to tease apart these effects with a comparative analysis relying on a sample encompassing multiple convergent acquisitions of the same lifestyle, covering as extensively as possible the body size range within each clade, and using subsets of data when lifestyles significantly differ in the represented species’ body size.

### Lifestyles, species, and tree

We defined specialised lifestyles as follows: fully aquatic, species that live exclusively in water [19]; aerial, species that are able to fly or glide [20]; and subterranean, species spending the greater part of their lives underground [21]. We regarded the remainder of the mammalian lifestyles, including semi-aquatic or arboreal taxa, as ‘non-specialised’. These four lifestyle classes were coded for all extant mammal genera (Fig. 1; no lifestyle variation was recognised within genera). The main timetree used for phylogenetically informed analyses was extracted from TimeTree.org [76].

Furthermore, we acquired bone structure data for all specialised clades, as well as for the most closely related taxa of each clade that can be regarded as terrestrial (which is distinct from the ‘non-specialised’ class defined above), i.e. not described as (semi-) aquatic, arboreal/scansorial, or fossorial. Taxa presenting these less specialised, non-terrestrial lifestyles were excluded from the sampling because of the potential effects these lifestyles can entail on bone structure. Primary data regarding non-specialised species’ lifestyles were taken from Nowack [77]. Finally, representatives of the other terrestrial families (defined with the same criteria) were also sampled, in an endeavour to cover the diversity of extant mammals. As a result, representatives of each specialised clades, their terrestrial sister groups (TSG), and of all other terrestrial families but two (Dinomyidae and Antilocapridae) were sampled (Additional file 1, Figure S3). We endeavoured to sample at least 5 specimens per specialised clade and its TSG. In some clades, represented by one or two species, that motivated the acquisition of repeats for the same species (e.g. the marsupial mole, *Notoryctes typhlops*). In addition to these extant species, we have extended the sampling of the sirenians (represented by two extant genera) with the recently extinct Steller’s sea cow (*Hydrodamalis gigas*), given that skeletal remains with a preservation comparable to that of extant species were available. This amounts to 190 museum specimens, representing 182 species (Additional file 3A). The main timetree was pruned to the subset of sampled species and missing species were added by hand: two species names were corrected (*Aotus azarai* = > *Aotus azarae* according to Wilson & Reeder [78]; *Tatera* sp. KIK1704 => *Tatera indica*); seven species names were swapped with other species of the same genus (simple renaming as only one species of each of these genera was sampled; *Rhizomys pruinosus* => *Rhizomys sumatrensis*; *Paraechinus aethiopicus* => *Paraechinus hypomelas*; *Petinomys setosus* => *Petinomys fuscocapilus*; *Abrocoma cinerea* => *Abrocoma budini*; *Hylomys parvus* => *Hylomys megalotis*; *Ctenomys torquatus* => *Ctenomys brasiliensis*; *Petaurillus kinlochii* => *Petaurillus hosei*); four species were added to the tree using external sources (*Anomalurus pelii*, divergence from most closely related sampled species *A. derbianus* at 12.2 Ma, [79]; *Idiurus macrotis*, divergence from *I. zenkeri* at 11.2 Ma, P.-H. Fabre, pers. comm.; *Oryzorictes tetradactylus*, divergence from most closely related sampled species *O. hova* at 5.13 Ma [80]; *Hydrodamalis gigas*, divergence from most closely related sampled species *Dugong dugon* at 28.59 Ma [81]).

The final, altered tree (182 species) can be found on figshare [104]. Lifestyle classification of all genera and timetree with 182 sampled species can also be found on figshare [104].

### Phenotypic data

**Fig. 6:**
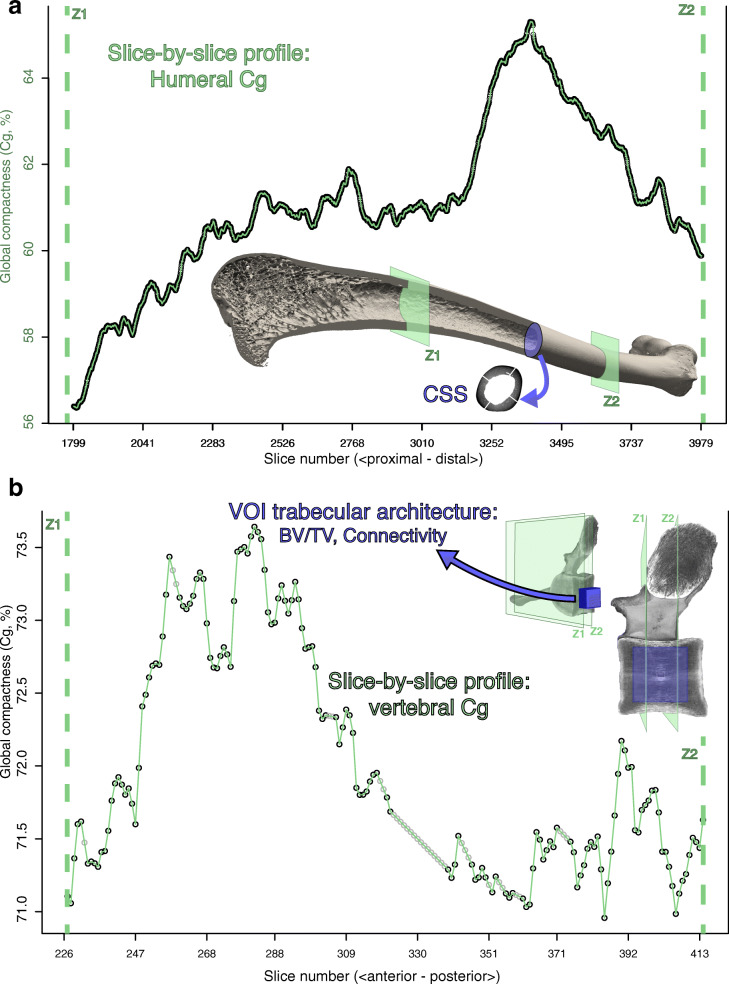
Description of the acquired bone structure parameters. Humeral (**a**) and vertebral (**b**) global compactness (Cg) were acquired along proximodistal and anteroposterior profiles, respectively. For the humerus (**a**), cross-sectional shape (CSS) at midshaft and diaphysis elongation (DE; diaphysis length/total cross-sectional area at midshaft^1/2^) were also acquired. For the vertebra, a volume of interest (VOI) was defined in the centre of the centrum, and the trabecular parameters bone fraction (BV/TV) and Connectivity were acquired. Example bones: humerus of a red fox (*Vulpes vulpes*, ZMB_Mam_49955 and lumbar vertebra of a dugong (*Dugong dugon* ZMB_Mam_69340); not to scale

Several traits were acquired to capture the bone structure properties of the whole humerus and whole mid-lumbar vertebra (in consistency with [38]). Micro-computed tomography (μCT) data were acquired for both skeletal elements. Scanning was performed with a Phoenix nanotom (General Electric GmbH Wunstorf, Germany), a FF35-CT-System (YXLON GmbH Hamburg, Germany), and HMX ST 225 (Nikon Metrology UK Ltd.). Scanning resolution was chosen to comply with the minimum relative resolution recommended for trabecular architecture analysis, often considered to be roughly five pixels per trabecula width (measured by dividing the mean trabecular thickness by the spatial resolution [82]). Scans acquired by Dumont et al. [38] were also used to expand the vertebral centrum dataset (see below). Prior to parameter acquisition, both the lumbar vertebra and the humerus were given a standard orientation using the software VG Studio Max 3.0–3.3 (Volume Graphics, Heidelberg, Germany; RRID:SCR_017997): the bones’ mediolateral and anteroposterior/proximodistal axes were aligned along the axes of the stack. All subsequent data acquisition was performed with the software Fiji/ImageJ [83] (2.0; RRID:SCR_002285; RRID:SCR_003070). We selected parameters to quantify the main mechanical constraints acting on the bones under study. While it is not possible to strictly compare humeral and vertebral structure due to their widely departing conformation, the mean Cg (global compactness; equivalent to bone fraction) was acquired in an analogous way. Many studies have quantified bone structure traits for long bones, and we focused on three parameters for the humerus: CSS (cross-sectional shape), DE (diaphysis elongation), and mean Cg, which can be argued to reflect direction of bending (be it uni- or multidirectional) or torsion for the former two, as well as compressive loads (terrestrial locomotion) and buoyancy (aquatic locomotion) for the latter [7, 10, 49, 84]. CSS—the ratio of maximum and minimum second moment of area (dimensionless parameter [85])—was acquired at midshaft (plug-ins *Optimise Threshold* and *Slice Geometry*, BoneJ v. 1.4 [86]). DE was defined as the ratio between the diaphyseal length of the humerus (here defined as 40% of the humerus’ functional length, see below) and the square root of the total cross-sectional area at midshaft (making this parameter dimensionless). Humeral mean Cg (equivalent to bone fraction; dimensionless parameter) was acquired using the approach described in Amson [87] (Fig. 6). In brief, slice-by-slice profiles are computed for each parameter of interest—here Cg and total cross-sectional area—along an anatomical axis. In this case, proximodistal profiles were computed for Cg in a region spanning the middle 40% of the humerus’ functional length (here assimilated to the greatest distance between proximal and distal articular surfaces), to ascertain that only the diaphysis would be sampled across all studied taxa and their disparate bone morphology (Fig. 6a). Mean Cg is then computed based on the mean value of all included slices. The total cross-sectional area was also measured in this region for its mean value to be used as a body size proxy (see below). Fewer studies have quantified bone structure traits for vertebrae. For biomechanical reasons, the centrum and its trabeculae were previously analysed in comparative studies [38]. But it became clear that such an approach would not suffice for our dataset, as many small mammals feature a simple centrum architecture with few trabeculae. We hence also measured mean Cg in the central region of the whole vertebrae (i.e. using slice-by-slice profiles comprising all those cross sections for which the vertebral canal is complete; Fig. 6b) to have an overall assessment of the vertebral robusticity. A similar approach based on single cross sections was used to investigate aquatic adaptations in snakes [88]. BoneJ was also used to quantify 3D trabecular architecture for cubic VOI defined to be as large as possible while being centred in the middle of the lumbar vertebra’s centrum [44]. VOIs here ranged from 0.016 mm^3^ (Hose’s pygmy flying squirrel, *Petaurillus hosei*) to 3.741E+ 05 mm^3^ (Steller’s sea cow, *Hydrodamalis gigas*; see 3a, c). One should bear in mind that size and shape of VOIs are known to impact parameter measurements [89, 90]. We consider that our approach is appropriate to sample functionally analogous regions in a dataset with such a wide range of body sizes. Trabecular parameters were acquired after binarization and purifications (removal of isolated particles of the extracted VOI stacks ;BoneJ plug-ins *Optimise Threshold* and *Purify*). Because few (if any) trabeculae were included in the VOI of some taxa (see Results), we refrained from further analysing trabecular parameters that would be spurious and focused on the VOI’s BV/TV (bone volume / total volume ratio; plug-in *Volume Fraction*; dimensionless parameter) and Connectivity (and the associated Connectivity density, Conn.D; plug-in *Connectivity*; approximates the number of trabeculae and this number per unit volume, respectively). For a regression of the form Conn. D [mm^− 3^] ~ body mass [g]^a^, the coefficient denoting isometry would be − 1 [74]. Because of this counterintuitive scaling, we chose to focus the analyses on Connectivity (Conn.D values are however reported with the other trabecular parameters and its scaling is shown in Additional file 1, Figure S2). The standard Trabecular Thickness (Tb.Th), Trabecular Spacing (Tb.Sp), Degree of Anisotropy (DA), and the main direction of the trabeculae (MDT; obtained with the R function cosap, Directional package [91] from the *x*-, *y*-, and *z*-components of the eigenvector defining the main orientation of the anisotropy; Additional file 5) were measured for VOIs with a Connectivity higher than 40 (threshold under which DA becomes inconsistent in this dataset). The anteroposterior length of the vertebral centrum was also measured to be used as a body size proxy.

Both humerus and lumbar vertebrae were not available for each sampled specimen. Furthermore, some of the CT scans of Dumont et al. [38] only captured the centrum, so only parameters of the centrum’s VOI could be acquired. Therefore, the analyses were run with three datasets: whole humerus (159 specimens), whole vertebra (160 specimens), and vertebral centrum (188 specimens). All specialised clades and outgroups were represented in all datasets. Parameters were averaged for those species that were represented by several specimens. All raw values, including scan absolute and relative resolutions [82], can be found on figshare [104]. Descriptive statistics are given in Additional file 3B.

In order to avoid potential bias affecting bone structure, specimens were selected to be adult (as indicated by size and/or epiphyseal fusion), devoid of apparent disease, and coming from the wild. But as marsupials and many small-sized mammals maintain unfused humeral epiphyses well into adulthood (if not throughout life), humeral epiphyses VOI parameters were not included in this analysis. Species body mass were taken from the AnAge [92] and MOM v1.4 [93] databases.

### Evolutionary history of lifestyle transitions in mammals

All data analyses were performed with R [94] (3.6.2; RRID:SCR_001905). A reconstruction of the ancestral character states was performed for the tree of extant mammal genera (1167 tips) using stochastic character mapping (make.simmap function, 1000 simulations, equal rate model; phytool package [95]).

### Size effect and lifestyle signal in bone structure parameters

All regressions and AN(C)OVAs were performed with generalised least squares linear models comprising a within-group correlation structure based on the optimised lambda value of the model’s residuals [96] (gls function, nlme package [97], corPagel function, APE package [98]). Nagelkerke pseudo *R*^2^ were computed with the rsquared function (piecewiseSEM package [99]). For AN(C)OVAs, post hoc pairwise comparisons were performed with the glht and mcp functions (Tukey’s multiple comparisons; multcomp package [100]). Variables were log10-transformed when their distribution was blatantly deviating from normality. For Connectivity, for which log10-transformation was recommendable, two specimens had a value of 0 (empty VOIs). Their values were hence replaced by the minimum Connectivity otherwise found in the dataset (0.875) subtracted by 10% to make the log10-transformation possible.

Body mass and specimen-specific size proxies were used to test the effect of body size on the parameters. For the latter, we used mean total cross-sectional area [7] (see above) for the humeral dataset, and centrum length [88] for the vertebral datasets. We only discuss the analyses that use body mass (above and Additional file 3C), as the ones performed with one of the proxies essentially yielded the same results. The latter are nevertheless detailed in Additional file 3D. While our sampling endeavoured to cover the whole range of body sizes for each lifestyle, a difference of size was found among them: aquatic taxa are larger than other lifestyles (*p* values < 0.014; see also Additional file 2). No conspicuous differences were found between the other lifestyles (*p* values > 0.194). Furthermore, the terrestrial taxa also feature a greater disparity in their sizes, their range encompassing all other lifestyles but the largest aquatic taxa. Subsets of the datasets for which the terrestrial class content was pruned to match the size and disparity of the other classes were therefore additionally examined: to compare subterranean and aerial lifestyles to the terrestrial one, the latter was pruned to 60 taxa for the humeral dataset (from the 83 original taxa), and to 60 and 62 taxa for the vertebral datasets (from the original 82 and 92 taxa); to compare the aquatic and terrestrial lifestyles, the latter was pruned to 7 taxa for the humeral dataset, and 9 and 14 taxa for the vertebral datasets.

We investigated the effect of size and lifestyle on each studied bone structure parameters using ANCOVAs. When a significant correlation between the size proxy and the parameter under study was recovered, residuals of the corresponding regression were computed. The latter were regarded as the size-corrected version of these parameters [101], which were used for the convergence analysis, as well as for visualisation purposes (phenograms and boxplots, see below). Using a body size proxy as a covariate does not mean here that we consider it as a nuisance parameter. Several of the investigated parameters are intrinsically dependent on size, and our approach is interested in departures from the scaling found in the non-specialised lifestyle.

### Convergence analysis

We quantified the convergent acquisition of each specialised lifestyle using the framework of the convevol package [102] for R. For each lifestyle, the number and composition of convergent clades was recovered from the result of the stochastic character mapping (Fig. 1). While the stochastic character mapping suggested that three genera of petauroids (gliding marsupials and their close relatives) acquired their aerial lifestyle independently (Additional file 1, Figure S1), all members of this clade are here classified as non-specialised but non-terrestrial (either arboreal or scansorial [103]). Because these other petauroids were not sampled (see sampling criteria above), only one convergence was counted for petauroids. Accordingly, the datasets were aggregated by taking the mean values of each converging clade, as well as each of their respective TSG. The timetree was correspondingly amended by reducing each specialised clade and each sister group to one tip (Additional file 1, Figure S4). We used univariate versions of the convevol functions to analyse the evolution of each trait of interest. We computed the convergence index C1 and associated *p* value (convratsig function), which gives an overall assessment of how much the phenotypes of converging clades evolved towards one another [102]. Because we studied the traits univariately, and because our sampling comprises a non-specialised sister group for each converging clade, we also investigated the direction of evolution in the last branch leading to each converging clade. We hence wrote a custom function convDir based on convevol’s convnum. For each converging tip, the function assesses whether its value is greater or lower than the reconstructed value of the node directly ancestral to it. The function also assesses whether the tip value falls outside the 95% confidence interval (95CI) of the reconstructed ancestral value. Phenograms (traitgrams) were plotted with the phenogram function (phytools package [95]). Modified convevol functions and convDir are available on GitHub: github.com//eliamson/convevol1d.

### Phylogenetic signal

To avoid the potential influence of lifestyles, only terrestrial species were included in this analysis. Pagel’s lambda (phylosig function, phytools package [95]) was computed for all terrestrial species taken individually and also after aggregating the TSG (as for the convergence analysis, taking the mean of each clade). This aggregation was used to assess the influence of the most recent diversifications on the phylogenetic signal. The size-corrected trait values (see above) were used when relevant (for all traits but CSS).

## Supplementary Information

**Additional file 1.** Additional figures 1-4.

**Additional file 2.** Distribution of body size according to lifestyle.

**Additional file 3.** Supplementary results. A, Specimen list; B, Descriptive statistics; C, AN(C)OVAs with body mass; D, AN(C)OVAs with specimen-specific body size proxies; Phylogenetic signal.

**Additional file 4.** Phenograms depicting the reconstructed evolution of each trait among specialised clades.

**Additional file 5.** Additional trabecular parameters of the vertebral centrum: a, Trabecular Thickness (Tb.Th); b, Trabecular Spacing (Tb.Sp); c, Degree of Anisotropy (DA), and d, the main direction of the trabeculae (MDT).

## Data Availability

Data: The datasets generated and analysed during the current study are available on figshare (doi: 10.6084/m9.figshare.12600440) [104]. Raw CT scan data related to the specimens of the Museum für Naturkunde (Berlin, Germany), the Naturhistorisches Museum Wien (Austria), and the Natural History Museum (NHMUK)—the bulk of the dataset—are curated by the former institution and available at 10.7479/gf2s-8a34 [105]. Raw data for additional specimens of the Institut royal des Sciences naturelles de Bruxelles (Belgium), Muséum national d’Histoire naturelle, Paris (France), and Zoologische Forschungsmuseum Alexander Koenig, Bonn (Germany) are curated by these institutions. **Code:** Univariate implementation of the functions of the R package convevol [102], as well as the custom function convDir, are available on GitHub: github.com/eliamson/convevol1d.
