# Supplementary material for: Differing effects of size and lifestyle on bone structure in mammals
Source: BMC Biol. 2021 Apr 29;19:87. doi: 10.1186/s12915-021-01016-1 (PMC8086358; doi:10.1186/s12915-021-01016-1)
Supplement: Supplementary file 1 — Additional file 1. Additional figures 1-4. [file 12915_2021_1016_MOESM1_ESM.pdf]

Supplementary Figures for:

Differing effects of size and lifestyle on bone structure in mammals

Eli Amson[1,]* & Faysal Bibi[1]

[1]Museum für Naturkunde, Leibniz-Institut für Evolutions- und Biodiversitätsforschung, Invalidenstraße 43, 10115 Berlin, Germany
*Corresponding author, eli.amson@mfn.berlin

Figure S1. **2**
Figure S2. **3**
Figure S3. **4**
Figure S4. **5**
References **5**

Figure S1.
Acquisitions of gliding lifestyle in Petauroidea. The most probable reconstructed ancestral state for Petauroidea is terrestrial (species tree from TimeTree.org [76]; make.simmap function of the phytools package [94], model with equal rates of transition, 1000 simulations), implying that an aerial lifestyle was convergently acquired in *Acrobates*, *Petauroides*, and *Petaurus*.

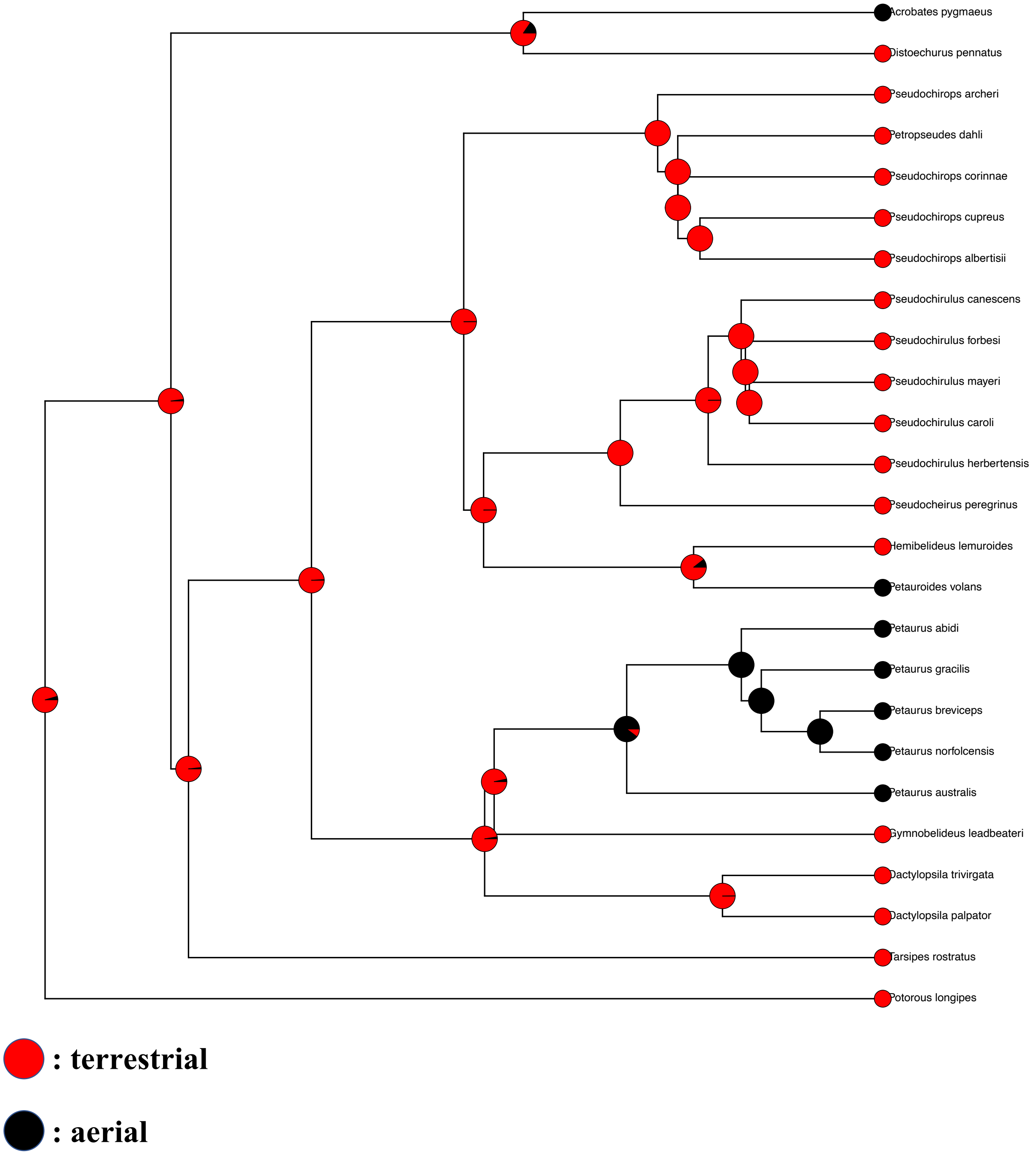

Figure S2.

Regression of the Connectivity Density (Conn.D; mm^{-3}) against body mass (g). A negative correlation is recovered: with log10-transformed variables, the regression coefficient is -0.34 (Standard error = 0.03). Note that this coefficient denotes positive allometry, because isometry for such a regression would be indicated by a slope of -1. Indeed, Connectivity Density has a dimension of a number per unit of volume. If the trabeculae would enlarge isometrically with increasing size, the same number of trabeculae would occupy a larger volume (so the ratio would be lower in larger species).

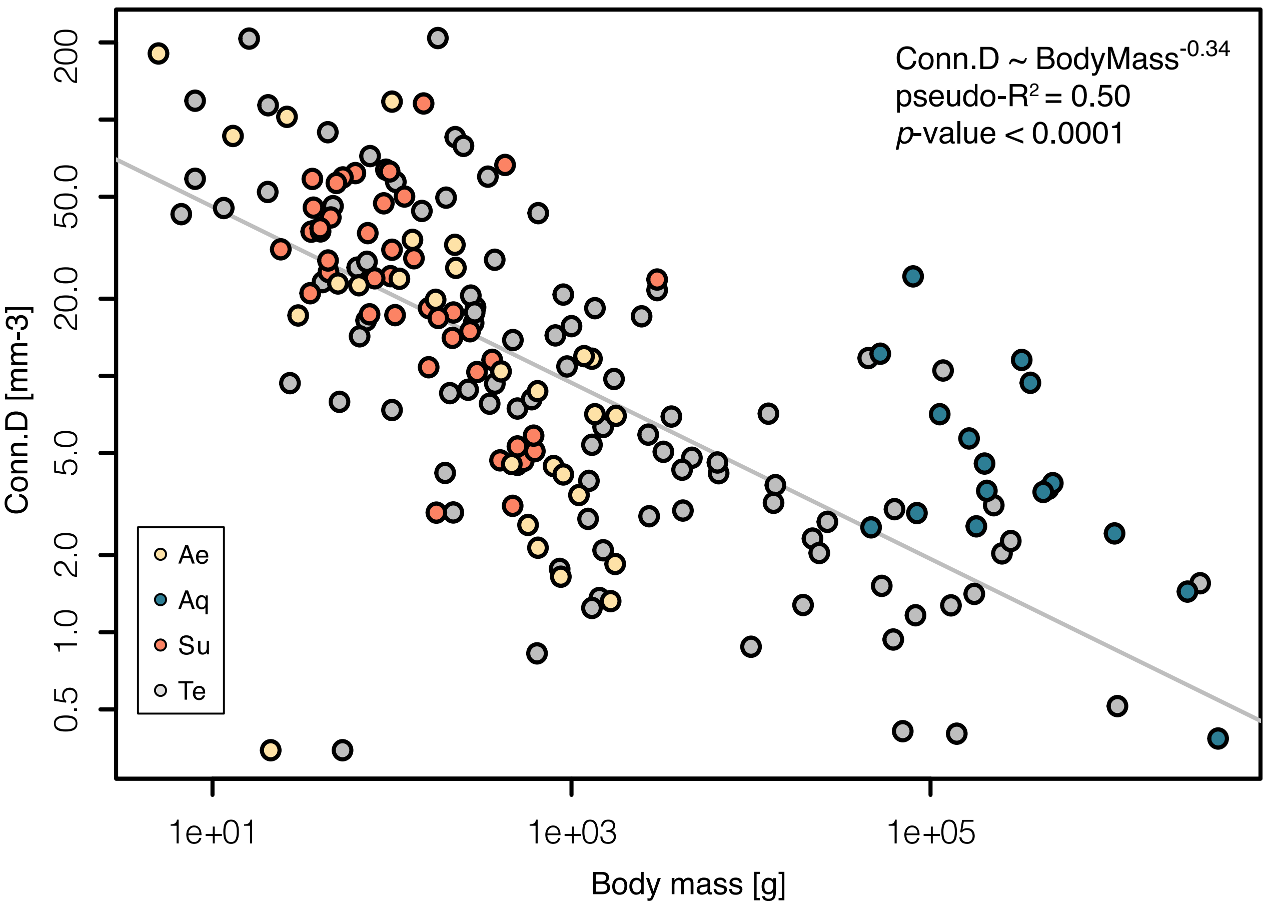

Figure S3.

Timetree of the mammalian species sampled. Colours correspond to the specialised lifestyles, i.e., aerial, aquatic, and subterranean; black corresponds to the terrestrial lifestyle (note that this is a subgroup of the class 'non-specialised' used for the Fig.1). States at the nodes are reconstructed with stochastic character mapping (make.simmap function, 1000 simulations, equal rate model; phytool, Revell 2012). Each silhouette represents an independent acquisition of one of the three specialised lifestyle. Abbreviations: Anom., Anomaluromorpha; Lag., Lagomorpha; Xe, Xenarthra.

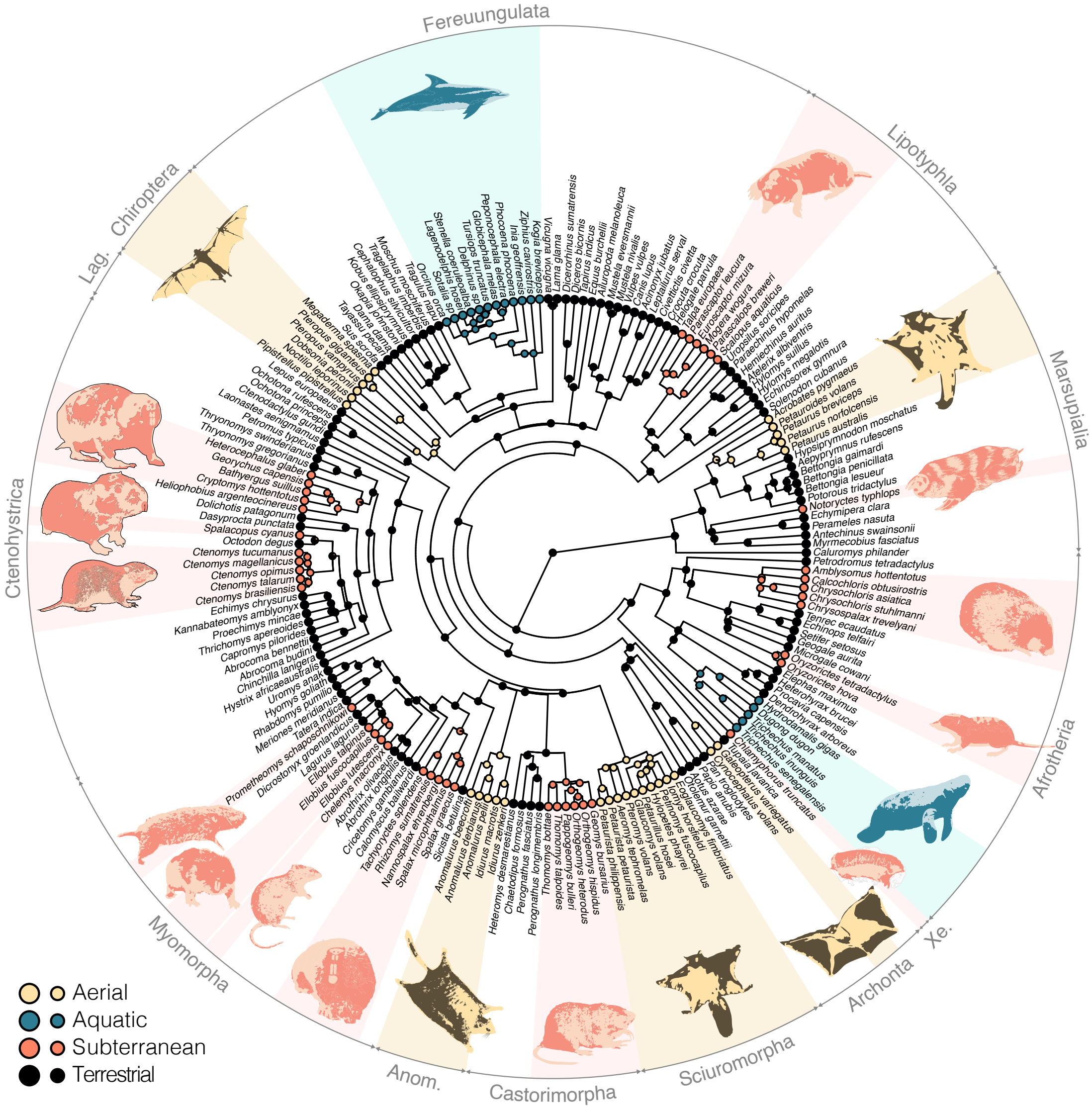

Figure S4.
Timetree amended by reducing each specialised clade and each sister-clade to one tip.

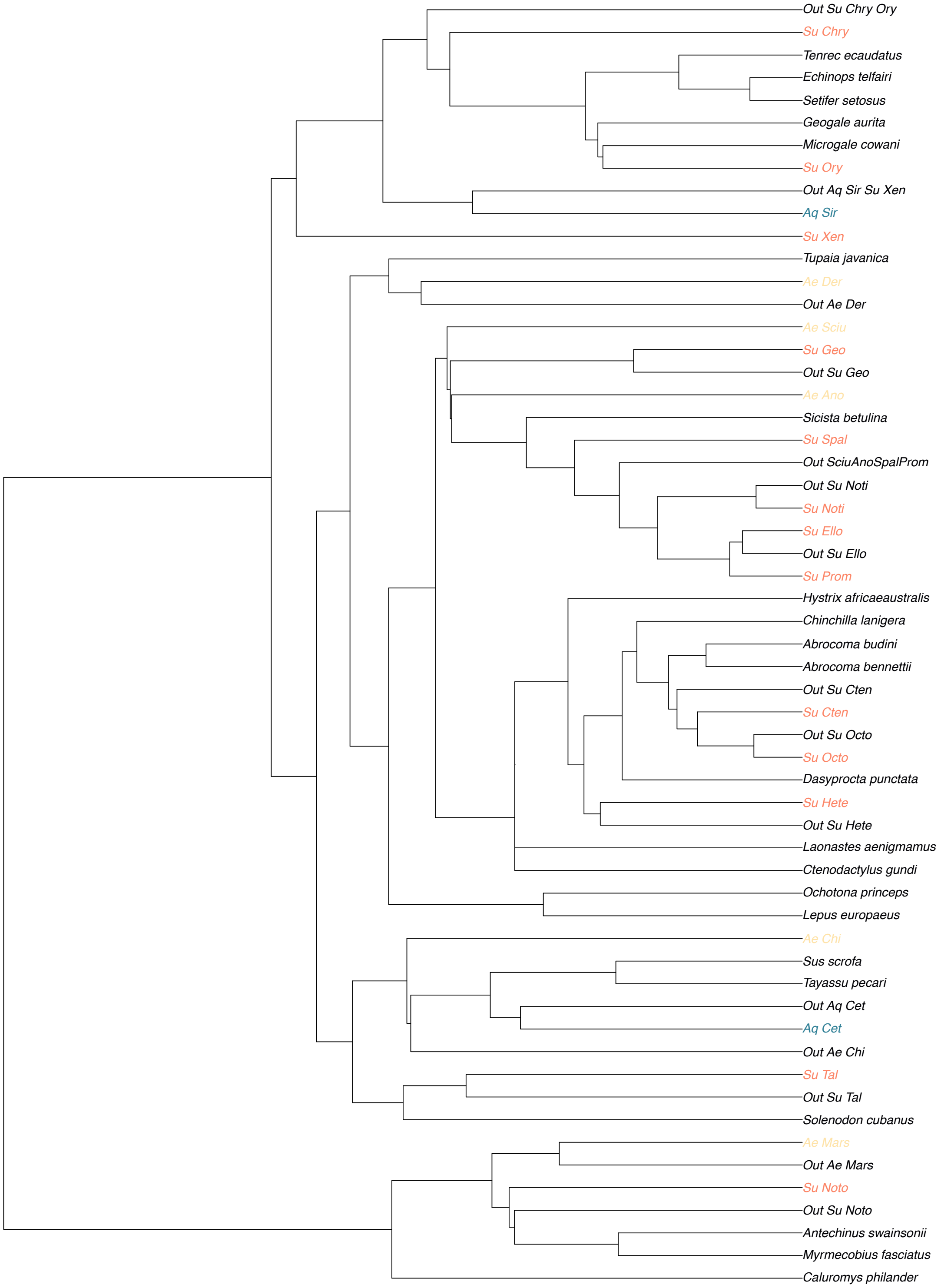

References

Lefcheck, J. S. 2016. piecewiseSEM: Piecewise structural equation modelling in R for ecology, evolution, and systematics. Methods in Ecology and Evolution 7:573–579.

Paradis, E., J. Claude, and K. Strimmer. 2004. APE: Analyses of phylogenetics and evolution in R language. Bioinformatics 20:289–290.

Pinheiro, J., D. Bates, S. DebRoy, D. Sarkar, and . R Core Team. 2016. nlme: Linear and nonlinear mixed effects models. R package.

Revell, L. J. 2010. Phylogenetic signal and linear regression on species data. Methods in Ecology and Evolution 1:319–329.

Revell, L. J. 2012. phytools: An R package for phylogenetic comparative biology (and other things). Methods in Ecology and Evolution 3:217–223.
