# Supplementary material for: Differing effects of size and lifestyle on bone structure in mammals
Source: BMC Biol. 2021 Apr 29;19:87. doi: 10.1186/s12915-021-01016-1 (PMC8086358; doi:10.1186/s12915-021-01016-1)
Supplement: Supplementary file 2 — Additional file 2. Distribution of body size according to lifestyle. [file 12915_2021_1016_MOESM2_ESM.pdf]

Additional File 2 for:

Differing effects of size and lifestyle on bone structure in mammals

Eli Amson[1,*] & Faysal Bibi[1]

[1]Museum für Naturkunde, Leibniz-Institut für Evolutions- und Biodiversitätsforschung, Invalidenstraße 43, 10115 Berlin, Germany
*Corresponding author, eli.amson@mfn.berlin

Distribution of body size according to lifestyle.

The log-transformed vertebral centrum length is used here as a size proxy. Abbreviations: Ae, Aerial; Aq, Aquatic; Su, Subterreanean; Te, terrestrial.

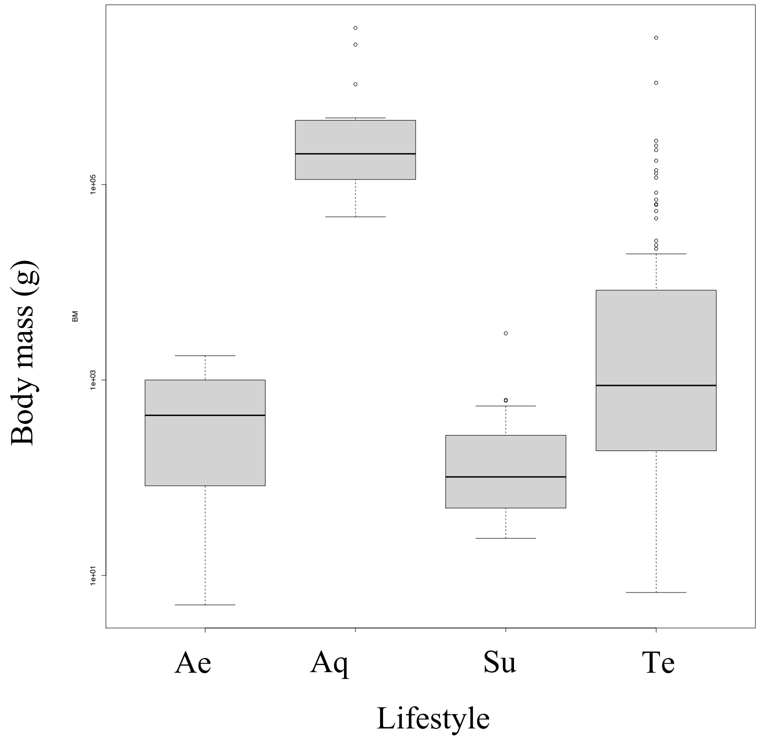

Result of post-hoc pairwise comparisons (glht function, multcomp package (Hothorn et al., 2008):

	Estimate	Std. Error	z value	Pr(>|z|)	
Aq - Ae == 0	2.3806	0.6636	3.587	0.00152	**
Su - Ae == 0	0.5747	0.4127	1.393	0.47116	
Te - Ae == 0	0.7363	0.3827	1.924	0.19390	
Su - Aq == 0	-1.8058	0.5838	-3.093	0.00902	**
Te - Aq == 0	-1.6443	0.5596	-2.938	0.01449	*
Te - Su == 0	0.1615	0.1852	0.872	0.80000	

Hothorn T, Bretz F, Westfall P. 2008. Simultaneous inference in general parametric models. *Biometrical J* **50**:346–363. doi:10.1002/bimj.200810425
