# Supplementary material for: Differing effects of size and lifestyle on bone structure in mammals
Source: BMC Biol. 2021 Apr 29;19:87. doi: 10.1186/s12915-021-01016-1 (PMC8086358; doi:10.1186/s12915-021-01016-1)

Additional File 3 for:

Differing effects of size and lifestyle on bone structure in mammals

Eli Amson[1,]* & Faysal Bibi[1]

[1]Museum für Naturkunde, Leibniz-Institut für Evolutions- und Biodiversitätsforschung, Invalidenstraße 43, 10115 Berlin, Germany
*Corresponding author, eli.amson@mfn.berlin

Additional File 3A. Specimen list.

List of all sampled specimens, with their corresponding lifestyle and abbreviated name of their specialised clade, terrestrial sister-group of a specialised clade, or more distantly related terrestrial clade (the latter are referred to as "Else"). See corresponding timetree as Supplementary Figure 2.

Species	Collection number	Lifestyle	Clade abbreviation
Anomalurus beecrofti	ZMB_Mam_36475	Ae	Ae_Ano
Anomalurus derbianus	ZMB_Mam_36344	Ae	Ae_Ano
Anomalurus pelii	ZMB_Mam_18404	Ae	Ae_Ano
Idiurus macrotis	ZMB_Mam_10088	Ae	Ae_Ano
Idiurus zenkeri	ZMB_Mam_22747	Ae	Ae_Ano
Dobsonia peronii	ZMB_Mam_66825	Ae	Ae_Chi
Megaderma spasma	ZMB_Mam_85644	Ae	Ae_Chi
Noctilio leporinus	ZMB_Mam_85657	Ae	Ae_Chi
Pipistrellus pipistrellus	ZMB_Mam_55128	Ae	Ae_Chi
Pteropus giganteus	MNHN_A_1251	Ae	Ae_Chi
Pteropus vampyrus	ZMB_Mam_88424	Ae	Ae_Chi
Galeopterus variegatus	ZMB_Mam_69096	Ae	Ae_Der
Cynocephalus volans	ZMB_Mam_69105	Ae	Ae_Der
Acrobates pygmaeus	ZMB_Mam_6241	Ae	Ae_Mars
Petauroides volans	ZMB_Mam_106893	Ae	Ae_Mars
Petaurus australis	MNHN-ZM-MO1883-1533	Ae	Ae_Mars
Petaurus breviceps	ZMB_Mam_104697	Ae	Ae_Mars
Petaurus norfolcensis	MNHN-ZM-MO1961-951	Ae	Ae_Mars
Aeromys tephromelas	NHMUK 1977.2886	Ae	Ae_Sciu
Eoglaucomys fimbriatus	NHMUK GERM 1721.a	Ae	Ae_Sciu
Glaucomys volans	MNHN_ZM_MO_1951_1023	Ae	Ae_Sciu
Hylopetes phayrei	NHMUK ZD 1994.199	Ae	Ae_Sciu
Iomys horsfieldi	NHMUK 1977.2885	Ae	Ae_Sciu
Petaurillus hosei	NHMUK ZD 1900.7.29.26	Ae	Ae_Sciu
Petaurista petaurista	ZMB_Mam_78560	Ae	Ae_Sciu
Petinomys fuscocapilus	NHMUK 1977.465	Ae	Ae_Sciu
Petaurista philippensis	ZMB_Mam_91292	Ae	Ae_Sciu
Pteromys volans	ZMB_Mam_78602	Ae	Ae_Sciu
Delphinus sp	ZMB_Mam_697.59	Aq	Aq_Cet
Globicephala melas	MNHN_LR_M_961	Aq	Aq_Cet
Inia geoffrensis	ZMB_Mam_41500	Aq	Aq_Cet
Kogia breviceps	MNHN_LR_M_1644	Aq	Aq_Cet

Lagenodelphis hosei	MNHN_LR_M1687	Aq	Aq_Cet
Orcinus orca	MNHN_1880_260	Aq	Aq_Cet
Peponocephala electra	MNHN_LR_M_103.08.076	Aq	Aq_Cet
Phocoena phocoena	MNHN_LR_M_958	Aq	Aq_Cet
Sotalia sp	ZMB_Mam_35828	Aq	Aq_Cet
Stenella coeruleoalba	MNHN_LR_M1848	Aq	Aq_Cet
Tursiops truncatus	MNHN_LR_M_1127	Aq	Aq_Cet
Ziphius cavirostris	MNHN_LR_M_942	Aq	Aq_Cet
Dugong dugon	ZMB_Mam_69340	Aq	Aq_Sir
Hydrodamalis gigas	MNHN_AC_1919-48	Aq	Aq_Sir
Trichechus manatus	ZMB_Mam_17377	Aq	Aq_Sir
Trichechus senegalensis	ZMB_Mam_69334	Aq	Aq_Sir
Trichechus inunguis	ZMB_Mam_35805	Aq	Aq_Sir
Amblysomus hottentotus	NMW26095	Su	Su_Chry
Amblysomus hottentotus	NMW7194	Su	Su_Chry
Calcochloris obtusirostris	ZMB_Mam_35173	Su	Su_Chry
Chrysochloris stuhlmanni	NHMUK 1934.4.1.8	Su	Su_Chry
Chrysochloris asiatica	ZMB_Mam_76897	Su	Su_Chry
Chrysochloris asiatica	NMW970	Su	Su_Chry
Chrysospalax trevelyani	NHMUK ZE 1951.11.14.4	Su	Su_Chry
Ctenomys brasiliensis	NHMUK ZD 1897.10.3.68	Su	Su_Cten
Ctenomys opimus	IRSNB 13028	Su	Su_Cten
Ctenomys talarum	NHMUK ZE 1968.8.20.1	Su	Su_Cten
Ctenomys tucumanus	ZMB_Mam_18180	Su	Su_Cten
Ctenomys magellanicus	ZMB_Mam_38821	Su	Su_Cten
Ellobius fuscocapillus	NMW12052	Su	Su_Ello
Ellobius lutescens	NMW20329	Su	Su_Ello
Ellobius talpinus	ZMB_Mam_78541	Su	Su_Ello
Orthogeomys heterodus	ZMB_Mam_106984	Su	Su_Geo
Orthogeomys hispidus	NHMUK ZD 1993.120	Su	Su_Geo
Pappogeomys bulleri	NHMUK 93.2.5.45	Su	Su_Geo
Thomomys bottae	ZMB_Mam_078534	Su	Su_Geo
Thomomys talpoides	NMW63141	Su	Su_Geo
Thomomys talpoides	NMW63144	Su	Su_Geo
Bathyergus suillus	ZMB_Mam_107241	Su	Su_Hete
Cryptomys hottentotus	NMW18413	Su	Su_Hete
Geomys bursarius	NHMUK 1384.a	Su	Su_Hete
Georychus capensis	NHMUK ZD 2019.218	Su	Su_Hete
Georychus capensis	ZMB_Mam_106888	Su	Su_Hete
Heterocephalus glaber	ZMB_Mam_79109	Su	Su_Hete
Heliophobius argenteocinereus	ZMB_Mam_107240	Su	Su_Hete
Chelemys macronyx	NMW43360	Su	Su_Noti
Notoryctes typhlops	NHMUK ZD 1981.1181	Su	Su_Noto
Notoryctes typhlops	MNHN-ZM-MO 1893-473	Su	Su_Noto
Notoryctes typhlops	ZMB_Mam_35694	Su	Su_Noto
Spalacopus cyanus	ZMB_Mam_8317	Su	Su_Octo
Oryzorictes hova	NHMUK ZD 1991.248	Su	Su_Ory
Oryzorictes hova	MNHN-ZM-MO 1985-1624	Su	Su_Ory
Oryzorictes tetradactylus	ZFMK MAM 1979.0155	Su	Su_Ory
Prometheomys schaposchnikowi	MNHN_ZM_2012_24	Su	Su_Prom

Nannospalax ehrenbergi	NMW65068	Su	Su_Spal
Rhizomys sumatrensis	ZMB_Mam_21249	Su	Su_Spal
Spalax graecus	NMW2158	Su	Su_Spal
Spalax microphthalmus	ZMB_Mam_78545	Su	Su_Spal
Tachyoryctes splendens	ZMB_Mam_72566	Su	Su_Spal
Euroscaptor mizura	ZMB_Mam_103981	Su	Su_Tal
Mogera wogura	ZMB_Mam_62455	Su	Su_Tal
Parascalops breweri	NMW62569	Su	Su_Tal
Parascaptor leucura	NHMUK ZE 1951.11.12.9	Su	Su_Tal
Scalopus aquaticus	NHMUK ZE 1958.3.11.7	Su	Su_Tal
Talpa europaea	ZMB_Mam_60682	Su	Su_Tal
Chlamyphorus truncatus	ZMB_Mam_6007	Su	Su_Xen
Abrocoma budini	NHMUK 1920.3.17.63	Te	Else
Abrocoma bennettii	NMW23394	Te	Else
Caluromys philander	ZMB_Mam_26760	Te	Else
Chinchilla lanigera	ZMB_Mam_81126	Te	Else
Ctenodactylus gundi	ZMB_Mam_71181	Te	Else
Dasyprocta punctata	ZMB_Mam_72483	Te	Else
Dolichotis patagonum	MNHN_AC_2000-827	Te	Else
Echinops telfairi	ZMB_Mam_71612	Te	Else
Geogale aurita	MNHN-ZM-MO1982-1001	Te	Else
Hystrix africaeaustralis	ZMB_Mam_70881	Te	Else
Laonastes aenigmamus	NHMUK ZD 1998.409	Te	Else
Lepus europaeus	ZMB_Mam_70801	Te	Else
Microgale cowani	ZMB_Mam_71614	Te	Else
Myrmecobius fasciatus	ZMB_Mam_3121	Te	Else
Elephas maximus	Confluences MHNL	Te	Else
Ochotona princeps	ZMB_Mam_93877	Te	Else
Ochotona rufescens	MNHN_CG2000-409	Te	Else
Antechinus swainsonii	IRSNB_21d	Te	Else
Setifer setosus	ZMB_Mam_44588	Te	Else
Sicista betulina	NMW29531	Te	Else
Solenodon cubanus	ZMB_Mam_2761	Te	Else
Sus scrofa	ZMB_Mam_7975	Te	Else
Tayassu pecari	ZMB_Mam_A.18.11	Te	Else
Tenrec ecaudatus	NMW2432	Te	Else
Tupaia javanica	ZMB_Mam_87169	Te	Else
Acinonyx jubatus	MNHN_AC_1998.1981	Te	Out_Ae_Chi
Ailuropoda melanoleuca	ZMB_Mam_17246	Te	Out_Ae_Chi
Canis lupus	MNHN_CG_1996.2499	Te	Out_Ae_Chi
Crocuta crocuta	ZMB_Mam_13295	Te	Out_Ae_Chi
Equus burchellii	ZMB_Mam_15963	Te	Out_Ae_Chi
Helogale parvula	ZMB_Mam_ 22986	Te	Out_Ae_Chi
Leptailurus serval	ZMB_Mam_58145	Te	Out_Ae_Chi
Mustela eversmannii	ZMB_Mam_12407	Te	Out_Ae_Chi
Mustela nivalis	ZMB_Mam_ 56731	Te	Out_Ae_Chi
Diceros bicornis	ZMB_Mam_32194	Te	Out_Ae_Chi
Tapirus indicus	ZMB_Mam_4950	Te	Out_Ae_Chi
Civettictis civetta	ZMB_Mam_68945	Te	Out_Ae_Chi
Vulpes vulpes	ZMB_Mam_49955	Te	Out_Ae_Chi

Dicerorhinus sumatrensis	ZMB_Mam_105847	Te	Out_Ae_Chi
Aotus azarae	ZMB_Mam_35793	Te	Out_Ae_Der
Otolemur garnettii	ZMB_Mam_5294	Te	Out_Ae_Der
Pan troglodytes	MNHN_AC_1950.194	Te	Out_Ae_Der
Papio anubis	ZMB_Mam_74869	Te	Out_Ae_Der
Aepyprymnus rufescens	ZMB_Mam_35059	Te	Out_Ae_Mars
Bettongia gaimardi	ZMB_Mam_60597	Te	Out_Ae_Mars
Bettongia lesueur	NHMUK ZD 1858.10.16.3	Te	Out_Ae_Mars
Bettongia penicillata	NHMUK ZD 1858.5.26.23	Te	Out_Ae_Mars
Hypsiprymnodon moschatus	ZMB_Mam_78469	Te	Out_Ae_Mars
Potorous tridactylus	MNHN-ZM-MO 1881-1148	Te	Out_Ae_Mars
Cephalophus silvicultor	MNHN_AC_1981.1023	Te	Out_Aq_Cet
Dama dama	ZMB_Mam_94752	Te	Out_Aq_Cet
Kobus ellipsiprymnus	MNHN_AC_1887.1237	Te	Out_Aq_Cet
Lama glama	NHMUK GERM 1860.e	Te	Out_Aq_Cet
Moschus moschiferus	ZMB_Mam_71049	Te	Out_Aq_Cet
Okapia johnstoni	MNHN_AC_1978.27	Te	Out_Aq_Cet
Tragelaphus imberbis	ZMB_Mam_48713	Te	Out_Aq_Cet
Tragulus napu	ZMB_Mam_A 14 08	Te	Out_Aq_Cet
Vicugna vicugna	ZMB_Mam_56204	Te	Out_Aq_Cet
Dendrohyrax arboreus	ZMB_Mam_21098	Te	Out_Aq_Sir_Su_Xen
Dendrohyrax arboreus	NHMUK 6.6.5.23	Te	Out_Aq_Sir_Su_Xen
Heterohyrax brucei	ZMB_Mam_21479	Te	Out_Aq_Sir_Su_Xen
Procavia capensis	ZMB_Mam_89416	Te	Out_Aq_Sir_Su_Xen
Calomyscus bailwardi	NMW18256	Te	Out_SciuAnoSpalProm
Cricetomys gambianus	ZMB_Mam_5371	Te	Out_SciuAnoSpalProm
Dicrostonyx groenlandicus	ZMB_Mam_78542	Te	Out_SciuAnoSpalProm
Hyomys goliath	ZMB_Mam_034358	Te	Out_SciuAnoSpalProm
Meriones meridianus	ZMB_Mam_43005	Te	Out_SciuAnoSpalProm
Rhabdomys pumilio	ZMB_Mam_86677	Te	Out_SciuAnoSpalProm
Tatera indica	ZMB_Mam_72793	Te	Out_SciuAnoSpalProm
Uromys anak	ZMB_Mam_34364	Te	Out_SciuAnoSpalProm
Petrodromus tetradactylus	ZMB_Mam_84912	Te	Out_Su_Chry_Ory
Capromys pilorides	ZMB_Mam_3202	Te	Out_Su_Cten
Thrichomys apereoides	ZMB_Mam_8283	Te	Out_Su_Cten
Echimys chrysurus	ZMB_Mam_8345	Te	Out_Su_Cten
Kannabateomys amblyonyx	ZMB_Mam_7259	Te	Out_Su_Cten
Proechimys mincae	ZMB_Mam_13543	Te	Out_Su_Cten
Lagurus lagurus	NHMUK ZE 1962.3.21.2	Te	Out_Su_Ello
Chaetodipus formosus	MNHN-ZM-MO 1914-35B	Te	Out_Su_Geo
Heteromys desmarestianus	ZFMK MAM 1981.1464	Te	Out_Su_Geo
Perognathus longimembris	NMW61838	Te	Out_Su_Geo
Perognathus fasciatus	ZMB_Mam_5071	Te	Out_Su_Geo
Petromus typicus	ZMB_Mam_105768	Te	Out_Su_Hete
Thryonomys swinderianus	ZMB_Mam_72392	Te	Out_Su_Hete
Thryonomys gregorianus	NHMUK 76.193	Te	Out_Su_Hete
Abrothrix olivaceus	MNHN-ZM-MO_1884-1293	Te	Out_Su_Noti
Abrothrix longipilis	NHMUK ZD 1858.9.6.5	Te	Out_Su_Noti
Echymipera clara	ZMB_Mam_15764	Te	Out_Su_Noto
Perameles nasuta	IRSNB_39	Te	Out_Su_Noto

Octodon degus	ZMB_Mam_8321	Te	Out_Su_Octo
Atelerix albiventris	ZMB_Mam_5810	Te	Out_Su_Tal
Echinosorex gymnura	ZMB_Mam_72232	Te	Out_Su_Tal
Hemiechinus auritus	NMW41180	Te	Out_Su_Tal
Hylomys megalotis	NHMUK ZD 1999.47	Te	Out_Su_Tal
Hylomys suillus	NHMUK ZE 1960.8.4.7	Te	Out_Su_Tal
Paraechinus hypomelas	NMW15184	Te	Out_Su_Tal
Uropsilus soricipes	NMW64409	Te	Out_Su_Tal

Footnotes. Lifestyles: Ae, aerial; Aq, fully aquatic; Su, subterranean, Te, Terrestrial. Clades: each specialised clade is referred to with the abbreviation of the corresponding lifestyle followed by a unique abbreviation; the terrestrial most closely related species of each specialised clade is referred to as "Out_*the abbreviation of the specialised clade* (note that some of these correspond to several specialised clades); Else, other, more distantly related terrestrial families. Museum collections: IRSNB, Institut royal des Sciences naturelles de Bruxelles; MHNL, Musée des Confluences, Lyon, France; MNHN, Muséum national d'Histoire naturelle, Paris, France; NHMUK, Natural History Museum, London, UK; NMW, Naturhistorisches Museum Wien, Austria; ZFMK MAM, Zoologische Forschungsmuseum Alexander Koenig, Bonn, Germany; ZMB_Mam, Mammals collection of the Museum für Naturkunde, Berlin.

Additional File 3B. Descriptive statistics

Descriptive statistics. Tb.Th, Tb.Sp, DA, and Conn.D were acquired on a subset of the specimens for which the Connectivity exceeded 40 (as they are not meaningful for volumes of interest comprising few or no trabeculae).

		Mean	Min	Max
Vertebral mean Cg (%)				
	Terrestrial	35.220	17.789	62.674
	Aerial	33.164	14.921	45.912
	Aquatic	62.425	58.249	71.983
	Subterranean	30.556	17.289	51.209
Connectivity (no units)				
	Terrestrial	4958.585	0.000	298141.750
	Aerial	19.134	0.000	98.125
	Aquatic	100620.699	3500.000	711702.375
	Subterranean	82.298	2.500	1191.625
BV.TV (no units)				
	Terrestrial	0.228	0.000	0.533
	Aerial	0.212	0.000	0.425
	Aquatic	0.423	0.309	0.703
	Subterranean	0.203	0.029	0.489
Humeral mean Cg (%)				
	Terrestrial	60.664	37.513	82.480
	Aerial	53.130	30.213	62.519
	Aquatic	78.030	49.205	99.619
	Subterranean	61.914	42.445	80.032
CSS (no units)				
	Terrestrial	2.070	1.121	5.252
	Aerial	1.268	1.031	1.660
	Aquatic	1.953	1.281	2.873
	Subterranean	3.157	1.242	10.837
DE (no units)				
	Terrestrial	4.819	3.065	7.026
	Aerial	7.200	5.336	9.924
	Aquatic	2.386	0.847	3.681
	Subterranean	3.364	1.162	5.715
Tb.Th (mm)				
	Terrestrial	0.261	0.064	1.777
	Aerial	0.153	0.108	0.189
	Aquatic	0.346	0.127	1.557

	Subterranean	0.118	0.059	0.209
Tb.Sp (mm)				
	Terrestrial	0.752	0.272	2.999
	Aerial	0.528	0.361	0.762
	Aquatic	0.477	0.313	0.814
	Subterranean	0.418	0.255	0.692
DA (no units)				
	Terrestrial	0.723	0.382	0.908
	Aerial	0.631	0.549	0.703
	Aquatic	0.745	0.503	0.866
	Subterranean	0.830	0.635	0.932
Conn.D (mm^{-3})				
	Terrestrial	14.546	0.402	208.006
	Aerial	10.203	6.989	11.948
	Aquatic	5.988	0.385	24.455
	Subterranean	41.919	5.088	115.576

Additional File 3C. AN(C)OVAs with body mass

AN(C)OVAs detailed outputs.

Lifestyle abbreviations: Ae, Aerial; Aq, Aquatic; Te, Terrestrial; Su, Subterranean.

1. Vertebral parameters

1.1. Whole dataset

Vertebral mean Cg ~ log10(Body Mass) + Lifestyle

Pagel's lambda
0.4920789

Coefficients:

	Value	Std.Error	t-value	p-value
(Intercept)	16.526634	3.499226	4.722940	0.0000
log10(BM)	7.242742	0.631763	11.464330	**0.0000**

Multiple Comparisons of Means: Tukey Contrasts

	Value	Std.Error	z-value	Pr(>|z|)
Aq - Ae	8.3201	3.9621	2.100	0.1374
Su - Ae	-0.8203	2.2286	-0.368	0.9810
Te - Ae	-1.7844	2.0045	-0.890	0.7930
Su - Aq	-9.1403	3.7020	-2.469	0.0570
Te - Aq	-10.1045	3.4217	-2.953	**0.0145**
Te - Su	-0.9641	1.4334	-0.673	0.8980

Nagelkerke pseudo R^2: 0.6762754

Vertebral mean Cg ~ log10(Body Mass)

	Value	Std.Error	t-value	p-value
(Intercept)	14.397094	3.385307	4.252818	0.0000
log10(BM)	7.760812	0.590105	13.151577	**0.0000**

Nagelkerke pseudo R^2: 0.6329046

---

log10(Connectivity) ~ log10(Body Mass) + Lifestyle

Pagel's lambda
0.3782865

Coefficients:

	Value	Std.Error	t-value	p-value
(Intercept)	-0.4336638	0.26574029 -	1.631908	0.1045
log10(BM)	0.6428774	0.0486077	13.225837	**0.0000**

Multiple Comparisons of Means: Tukey Contrasts

	Value	Std.Error	t-value	p-value
Aq - Ae	1.4042	0.2840	4.943	**< 0.001**
Su - Ae	0.6612	0.1793	3.688	**0.00113**
Te - Ae	0.2756	0.1607	1.716	0.29370
Su - Aq	-0.7430	0.2652	-2.801	**0.02347**
Te - Aq	-1.1285	0.2346	-4.811	**< 0.001**
Te - Su	-0.3855	0.1214	-3.175	**0.00720**

Nagelkerke pseudo R^2: 0.769842

log10(Connectivity) ~ log10(Body Mass

	Value	Std.Error	t-value	p-value
(Intercept)	-0.5254737	0.9220687	-0.569886	0.5695
log10(BM)	0.6997265	0.0532410	13.142635	**0.0000**

Nagelkerke pseudo R^2: 0.6332184

BV.TV ~ log10(Body Mass) + Lifestyle

Pagel's lambda
0.1268139

Coefficients

	Value	Std.Error	t-value	p-value
(Intercept)	0.07216842	0.02955185	2.442095	0.0156
log10(BM)	0.05885153	0.00694925	8.468757	**0.0000**

Multiple Comparisons of Means: Tukey Contrasts

	Value	Std.Error	t-value	p-value
Aq - Ae	0.015705	0.036799	0.427	0.971
Su - Ae	-0.000248	0.023458	-0.011	1.000
Te - Ae	-0.029485	0.021057	-1.400	0.477
Su - Aq	-0.015953	0.035654	-0.447	0.967
Te - Aq	-0.045190	0.030415	-1.486	0.424
Te - Su	-0.029237	0.017833	-1.640	0.336

Nagelkerke pseudo R^2: 0.4737552

BV.TV ~ log10(Body Mass)

	Value	Std.Error	t-value	p-value
(Intercept)	0.05929340	0.02915428	2.033781	0.0435
log10(BM)	0.05840329	0.00604638	9.659215	**0.0000**

Nagelkerke pseudo R^2: 0.4389989

1.2. Terrestrial pruned to match Aquatic size

Vertebral Mean Cg ~ log10(Body Mass) + Lifestyle

Pagel's lambda
0.1980319

Coefficients

	Value	Std.Error	t-value	p-value
(Intercept)	14.439071	21.166159	0.682177	0.5071
log10(BM)	9.144339	3.987814	2.293071	**0.0392**
Te-Aq	-10.591180	3.049641 -	3.472927	**0.0041**

log10(Connectivity) ~ log10(Body Mass) + Lifestyle

Pagel's lambda
0.3864759

Coefficients

	Value	Std.Error	t-value	p-value
(Intercept)	-0.1833932	0.3345509 -	0.548177	0.5848
log10(BM)	0.5512236	0.1017805	5.415810	**0.0000**
Te-Aq	1.07108	0.24897	-4.302	**< 0.001**

1.3. Terrestrial pruned to match Aerial and Subterranean Size

log10(Connectivity) ~ log10(Body Mass) + Lifestyle

Pagel's lambda
0.3019896

Coefficients

	Value	Std.Error	t-value	p-value
(Intercept)	-0.0826887	0.2673884	-0.309246	0.7576
log10(BM)	0.4880283	0.0719708	6.780925	**0.0000**
Te-Ae	0.2647	0.1476	1.793	0.25427
Te-Su	-0.3803	0.1141	-3.334	**0.00397**

2. Humeral parameters

2.1. Whole dataset

Humeral mean Cg ~ log10(Body Mass) + Lifestyle

Pagel's lambda

0.6574772

Coefficients

	Value	Std.Error	t-value	p-value
(Intercept)	48.20127	5.661718	8.513543	0.0000
log10(BM)	2.91733	0.898195	3.247990	**0.0014**

Multiple Comparisons of Means: Tukey Contrasts

	Value	Std.Error	t-value	p-value
Aq - Ae	13.128	6.276	2.092	0.138
Su - Ae	6.036	3.415	1.768	0.263
Te - Ae	4.343	3.088	1.406	0.465
Su - Aq	-7.092	5.763	-1.231	0.578
Te - Aq	-8.785	5.428	-1.619	0.339
Te - Su	-1.694	2.009	-0.843	0.817

Humeral mean Cg ~ log10(Body Mass)

	Value	Std.Error	t-value	p-value
(Intercept)	50.89430	5.710878	8.911817	**0.000**
log10(BM)	3.56231	0.851195	4.185066	**0.000**

Nagelkerke pseudo R^2: 0.1398405

---

log10(CSS) ~ log10(Body Mass) + Lifestyle

Pagel's lambda
0.637239

Coefficients

	Value	Std.Error	t-value	p-value
(Intercept)	0.09745506	0.10245958	0.951156	0.3431
log10(BM)	0.01028903	0.01654009	0.622066	0.5349

Multiple Comparisons of Means: Tukey Contrasts

	Value	Std.Error	t-value	p-value
Aq - Ae	0.19283	0.11489	1.678	0.30715
Su - Ae	0.31182	0.06233	5.003	**< 0.001**
Te - Ae	0.20654	0.05634	3.666	**0.00119**
Su - Aq	0.11899	0.10569	1.126	0.64685
Te - Aq	0.01371	0.09946	0.138	0.99894
Te - Su	-0.10529	0.03700	-2.845	**0.01954**

log10(CSS) ~ log10(Body Mass)

	Value	Std.Error	t-value	p-value
(Intercept)	0.29166734	0.13393460	2.1776849	0.0310
log10(BM)	0.01243528	0.01739865	0.7147265	0.4759

Nagelkerke pseudo R^2: 0.005091859

---

log10(DE) ~ log10(Body Mass) + Lifestyle

Pagel's lambda
0.9782044

Coefficients

	Value	Std.Error	t-value	p-value
(Intercept)	0.8705816	0.08513065	10.226418	0.0000
log10(BM)	-0.0100423	0.00899478 -	1.116464	**0.266**

Multiple Comparisons of Means: Tukey Contrasts

	Value	Std.Error	t-value	p-value
Aq - Ae	-0.54516	0.07914	-6.888	**< 0.001**
Su - Ae	-0.31264	0.04427 -	7.063	**< 0.001**
Te - Ae	-0.19787	0.04121	-4.801	**< 0.001**
Su - Aq	0.23252	0.07019	3.313	**0.00443**
Te - Aq	0.34729	0.06781	5.122	**< 0.001**
Te - Su	0.11477	0.01987	5.776	**< 0.001**

log10(DE) ~ log10(Body Mass)

	Value	Std.Error	t-value	p-value
(Intercept)	0.7004276	0.10652242	6.575401	0.0000
log10(BM)	-0.0235471	0.01017686 -	2.313793	**0.022**

Nagelkerke pseudo R^2: 0.02494447

2.2. Terrestrial pruned to match Aerial and Subterranean Size

log10(CSS) ~ log10(Body Mass) + Lifestyle

Pagel's lambda
0.6403116

Coefficients

	Value	Std.Error	t-value	p-value
(Intercept)	0.0445801	0.11493737	0.387864	0.6988
log10(BM)	0.0316706	0.02457316	1.288830	0.1999
Te-Ae	0.22123	0.05966	3.708	**<0.001**
Te-Su	-0.10536	0.03875	-2.719	**0.0278**

log10(DE) ~ log10(Body Mass) + Lifestyle

Pagel's lambda
0.9773708

Coefficients

	Value	Std.Error	t-value	p-value
(Intercept)	0.8393310	0.08583106	9.778873	0.000
log10(BM)	0.0039557	0.01135413	0.348396	0.7281
Te-Ae	-0.20131	0.04143	-4.859	**<0.001**
Te-Su	0.11737	0.01973	5.949	**< 0.001**

2.3. DE allometry in the terrestrial taxa

log10(DE) ~ log10(Body Mass)

	Value	Std.Error	t-value	p-value
(Intercept)	0.7657001	0.05213702	14.686303	0.0000
log10(BM)	-0.0348112	0.00840933	-4.139593	**< 0.001**

Nagelkerke pseudo R^2: 0.2410223

Additional File 3D. AN(C)OVAs with specimen-specific body size proxies

AN(C)OVAs detailed outputs.

Lifestyle abbreviations: Ae, Aerial; Aq, Aquatic; Te, Terrestrial; Su, Subterranean.

1. Vertebral parameters

1.1. Whole dataset

Vertebral mean Cg ~ log(Vertebral_centrum_length) + Lifestyle

Pagel's lambda
0.5515288

Coefficients:

	Value	Std.Error	t-value	p-value
(Intercept)	15.967524	3.752081	4.255645	0.0000
log(VertL)	9.362762	0.825957	11.335656	**0.0000**

Multiple Comparisons of Means: Tukey Contrasts

	Value	Std.Error	z-value	Pr(>|z|)
Aq - Ae	10.6641	4.0266	2.648	**0.03541**
Su - Ae	0.4067	2.3187	-0.175	0.99786
Te - Ae	-1.8826	2.0900	-0.901	0.78737
Su - Aq	-11.0708	3.7476	-2.954	**0.01438**
Te - Aq	-12.5466	3.4651	-3.621	**0.00141**
Te - Su	-1.4758	1.4645	-1.008	0.72439

Vertebral mean Cg ~ log(Vertrebral_centrum_length)

	Value	Std.Error	t-value	p-value
(Intercept)	14.10409	4.159763	3.390599	9e-04
log(VertL)	10.02336	0.823731	12.168250	0.0000

Nagelkerke pseudo R^2: 0.5869862

---

log(Connectivity) ~ log(Vertebral_centrum_length) + Lifestyle

Pagel's lambda
0.55818

Coefficients:

	Value	Std.Error	t-value	p-value
(Intercept)	-0.760339	0.7991580	-0.951425	0.3427
log(VertL)	1.771254	0.1602438	11.053496	**0.0000**

Multiple Comparisons of Means: Tukey Contrasts

	Value	Std.Error	t-value	p-value
Aq - Ae	3.6395	0.7825	4.651	**< 0.001**
Su - Ae	1.5695	0.4958	3.165	**0.00736**
Te - Ae	0.6036	0.4470	1.350	0.50519
Su - Aq	-2.0701	0.7179	-2.883	**0.01831**
Te - Aq	-3.0360	0.6452	-4.706	**< 0.001**
Te - Su	-0.9659	0.3133	-3.083	**0.00969**

log(Connectivity) ~ log(Vertebral_centrum_length)

	Value	Std.Error	t-value	p-value
(Intercept)	-0.1872968	1.0885475 -	0.172061	0.8636
log(VertL)	1.8862857	0.1711185	11.023273	**0.0000**

Nagelkerke pseudo R^2: 0.4824922

BV.TV ~ log(Vertrebral_centrum_length) + Lifestyle

Pagel's lambda
0.3461863

Coefficients

	Value	Std.Error	t-value	p-value
(Intercept)	0.08123194	0.03981464	2.040253	0.0428
log(VertL)	0.07407308	0.00958539	7.727704	**0.0000**

Multiple Comparisons of Means: Tukey Contrasts

	Value	Std.Error	t-value	p-value
Aq - Ae	0.0233287	0.0422055	0.553	0.941
Su - Ae	-0.0009192	0.0272091	-0.034	1.000
Te - Ae	-0.0337508	0.0244241	-1.382	0.487
Su - Aq	-0.0242480	0.0396518	-0.612	0.922
Te - Aq	-0.0570795	0.0347457	-1.643	0.333
Te - Su	-0.0328316	0.0188409	-1.743	0.281

BV.TV ~ log(Vertebral_centrum_length)

	Value	Std.Error	t-value	p-value
(Intercept)	0.06783887	0.04113441	1.649200	0.1009
log(VertL)	0.07307459	0.00891487	8.196933	**0.0000**

Nagelkerke pseudo R^2: 0.3778663

1.2. Terrestrial pruned to match Aquatic size

Vertebral Mean Cg ~ log(Vertebral_centrum_length) + Lifestyle

Pagel's lambda

0.6178141

Coefficients

	Value	Std.Error	t-value	p-value
(Intercept)	10.456846	5.730015	1.824925	0.0716
log(VertL)	10.607906	1.268990	8.359330	**0.0000**
Te-Aq	-13.363764	3.322355	-4.022377	**0.0001**

log(Connectivity) ~ log(Vertebral_centrum_length) + Lifestyle

Pagel's lambda
0.5547799

Coefficients

	Value	Std.Error	t-value	p-value
(Intercept)	1.7962133	1.2443517	1.443493	0.1518
log(VertL)	1.4809046	0.2659399	5.568568	**0.0000**
Te-Aq	-2.8420086	0.6676666	-4.256629	**0.0000**

1.3. Terrestrial pruned to match Aerial and Subterranean Size

log(Connectivity) ~ log(Vertebral_centrum_length) + Lifestyle

Pagel's lambda
0.4313344

Coefficients

	Value	Std.Error	t-value	p-value
(Intercept)	0.731928	0.6578026	1.112687	0.2679
log(VertL)	1.252803	0.2184246	5.735632	**0.0000**
Te-Ae	0.525197	0.4018929	1.306808	0.1936
Te-Su	-0.995753	0.2857582	-3.484600	**0.0007**

2. Humeral parameters

2.1. Whole dataset

Humeral mean Cg ~ log(mean_total_cross-sectional_area) + Lifestyle

Pagel's lambda
0.64127

Coefficients

	Value	Std.Error	t-value	p-value
(Intercept)	52.27987	5.378621	9.719939	0.0000
log(MeanArea)	1.32066	0.553781	2.384808	**0.0184**

Multiple Comparisons of Means: Tukey Contrasts

	Value	Std.Error	t-value	p-value
Aq - Ae	15.7933	6.2245	2.537	**0.047**
Su - Ae	5.9057	3.4534	1.710	0.291
Te - Ae	5.0618	3.0864	1.640	0.328
Su - Aq	-9.8876	5.6649	-1.745	0.274
Te - Aq	-10.7315	5.4111	-1.983	0.174
Te - Su	-0.8439	2.0089	-0.420	0.972

Humeral mean Cg ~ log(mean_total_cross-sectional_area)

	Value	Std.Error	t-value	p-value
(Intercept)	56.06108	5.422449	10.338702	**0.000**
log(MeanArea)	1.79860	0.537809	3.344315	**0.001**

Nagelkerke pseudo R^2: 0.09309527

---

log(CSS) ~ log(mean_total_cross-sectional_area) + Lifestyle

Pagel's lambda
0.6544086

Coefficients

	Value	Std.Error	t-value	p-value
(Intercept)	0.2291447	0.22828617	1.003761	0.3171
log(MeanArea)	0.0302043	0.02320436	1.301667	<u>0.1951</u>

Multiple Comparisons of Means: Tukey Contrasts

	Value	Std.Error	t-value	p-value
Aq - Ae	0.39033	0.26194	1.490	0.41350
Su - Ae	0.69558	0.14557	4.778	**< 0.001**
Te - Ae	0.46138	0.13016	3.545	**0.00174**
Su - Aq	0.30525	0.23818	1.282	0.54551
Te - Aq	0.07106	0.22756	0.312	0.98811
Te - Su	-0.23419	0.08422	-2.781	**0.02354**

log(CSS) ~ log(mean_total_cross-sectional_area)

	Value	Std.Error	t-value	p-value
(Intercept)	0.6431275	0.29040728	2.214571	0.0283
log(MeanArea)	0.0421687	0.02446698	1.723495	<u>0.0869</u>

Nagelkerke pseudo R^2: 0.02652733

---

log(DE) ~ log(mean_total_cross-sectional_area) + Lifestyle

Pagel's lambda
0.9732087

Coefficients

	Value	Std.Error	t-value	p-value
(Intercept)	2.0175333	0.18358951	10.989371	0.0000
log(MeanArea)	-0.0419341	0.01234671 -	3.396376	**9e-04**

Multiple Comparisons of Means: Tukey Contrasts

	Value	Std.Error	t-value	p-value
Aq - Ae	-1.15655	0.17342	-6.669	**< 0.001**
Su - Ae	-0.68674	0.09803	-7.006	**< 0.001**
Te - Ae	-0.42660	0.09062	-4.708	**< 0.001**
Su - Aq	0.46981	0.15338	3.063	**0.00976**
Te - Aq	0.72995	0.14886	4.904	**< 0.001**
Te - Su	0.26014	0.04426	5.878	**< 0.001**

log(DE) ~ log(mean_total_cross-sectional_area)

	Value	Std.Error	t-value	p-value
(Intercept)	1.6223822	0.2285688	7.098002	0.0000
log(MeanArea)	-0.0627009	0.0144669	-4.334094	0.0000

Nagelkerke pseudo R^2: 0.085116

2.2. Terrestrial pruned to match Aerial and Subterranean Size

log(CSS) ~ log(mean_total_cross-sectional_area) + Lifestyle

Pagel's lambda
0.5932886

Coefficients

	Value	Std.Error	t-value	p-value
(Intercept)	0.6612625	0.20331205	3.252451	0.0015
log(MeanArea)	0.0640891	0.03335165	1.921618	0.0569
Te-Ae	-0.5146113	0.12943284 -	-3.975894	**0.0001**
Te-Su	0.2052093	0.08697299	-2.359460	**0.0198**

log(DE) ~ log(mean_total_cross-sectional_area) + Lifestyle

Pagel's lambda
0.9837882

Coefficients

	Value	Std.Error	t-value	p-value

(Intercept)	1.5611948	0.17930662	8.706844	0.000
log(MeanArea)	-0.0277517	0.01609240 -	1.724522	0.087
Te-Ae	-0.4323139	0.09628532	-4.489925	**0.000**
Te-Su	0.2558572	0.04572239	5.595885	**0.000**

Additional File 3E. Phylogenetic signal.
Phylogenetic signal among terrestrial species.

Sample size range from 82 to 92 (depending on the trait), for all species analysed individually and is 37 when terrestrial sister-groups (TSG) are aggregated. Mapping (function contmap, phytools package (Revell 2012) for the Connectivity and diaphysis elongation (DE), the only traits for which a significant signal was found when the TSG are aggregated, is also displayed.

Vertebral mean Cg (size-corrected)
All species individually
Phylogenetic signal lambda: 0.266709
logL(lambda) : -275.644
LR(lambda=0) : 2.3316
P-value (based on LR test) : 0.126772
TSG aggregated
Phylogenetic signal lambda : 7.07461e-05
logL(lambda) : -121.226
LR(lambda=0) : -0.0010767
P-value (based on LR test) : 1

BV.TV (size-corrected)
All species individually
Phylogenetic signal lambda : 0.124471
logL(lambda) : 90.5531
LR(lambda=0) : 1.64627
P-value (based on LR test) : 0.199467
TSG aggregated
Phylogenetic signal lambda : 7.07461e-05
logL(lambda) : 35.8722
LR(lambda=0) : -0.00101486
P-value (based on LR test) : 1

Connectivity (size-corrected)
All species individually
Phylogenetic signal lambda : 0.54035
logL(lambda) : -84.3502
LR(lambda=0) : 7.27064
P-value (based on LR test) : **0.00700909**
TSG aggregated
Phylogenetic signal lambda : 0.742712
logL(lambda) : -30.5181
LR(lambda=0) : 6.76367
P-value (based on LR test) : **0.0093032**

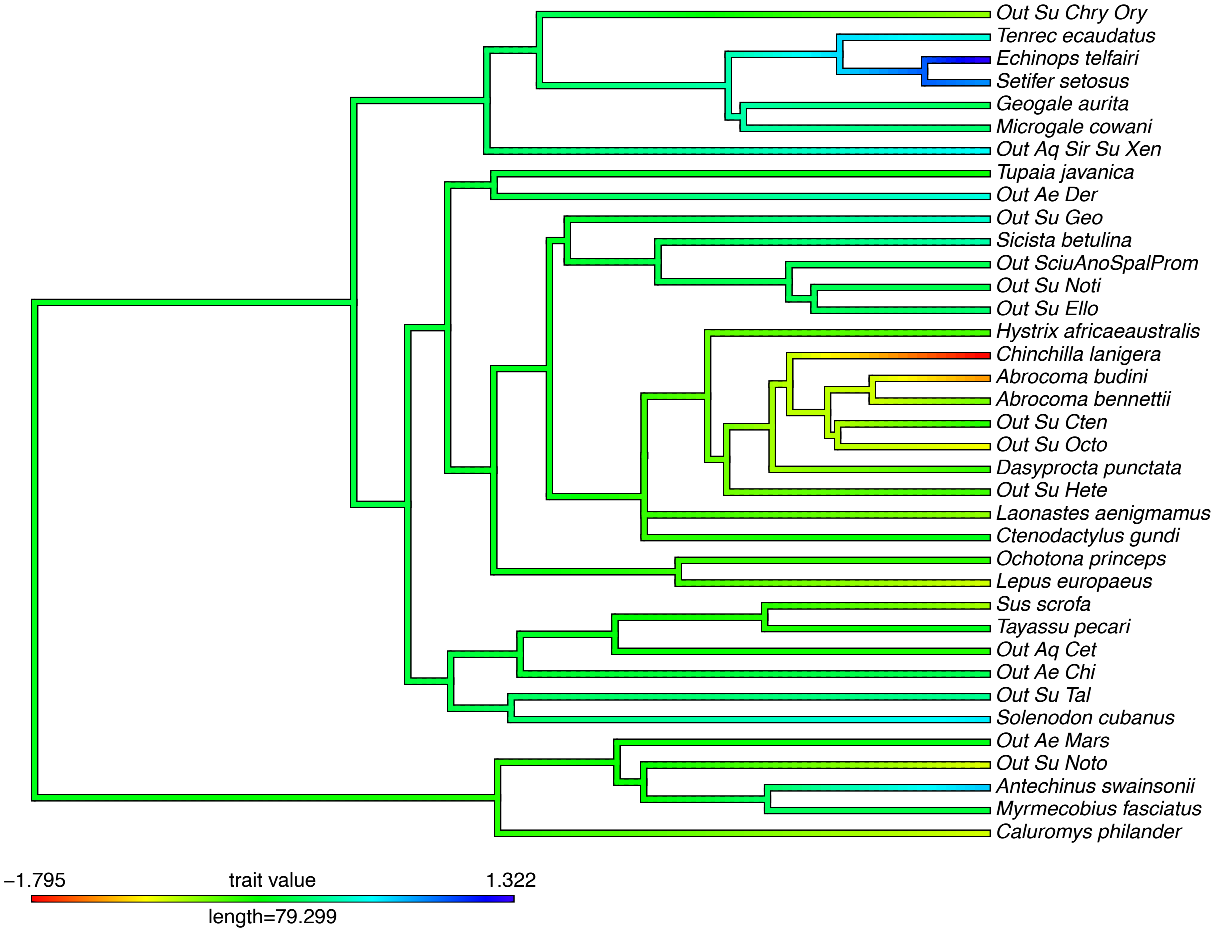

Humeral mean Cg (size-corrected)

<u>All species individually</u>

Phylogenetic signal lambda : 0.649341
logL(lambda) : -295.02
LR(lambda=0) : 10.8479
P-value (based on LR test) : **0.000989104**

<u>TSG aggregated</u>

Phylogenetic signal lambda : 7.07461e-05
logL(lambda) : -126.236
LR(lambda=0) : -0.000901852
P-value (based on LR test) : 1

log10(CSS)

<u>All species individually</u>

Phylogenetic signal lambda: 0.465252
logL(lambda): 49.6645
LR(lambda=0): 11.6607
P-value (based on LR test): **0.000638344**

scConnectivity

TSG aggregated

Phylogenetic signal lambda: 0.115859
logL(lambda): 19.9334
LR(lambda=0): 0.12607
P-value (based on LR test): 0.722542

DE (size-corrected)

All species individually

Phylogenetic signal lambda : 0.854163
logL(lambda) : 108.255
LR(lambda=0) : 19.0383
P-value (based on LR test) : **1.2812e-05**

TSG aggregated

Phylogenetic signal lambda : 0.792325
logL(lambda) : 51.7664
LR(lambda=0) : 4.13848
P-value (based on LR test) : **0.0419188**

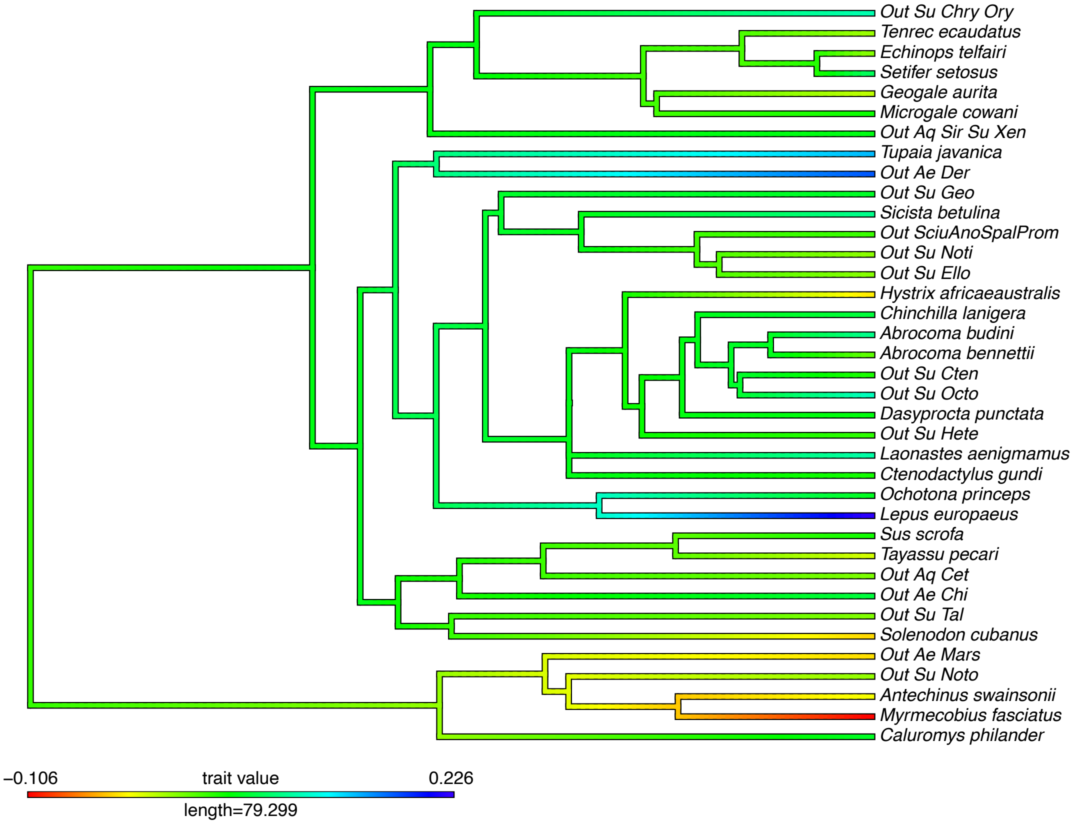

Supplement: Supplementary file 3 — Additional file 3. Supplementary results. A, Specimen list; B, Descriptive statistics; C, AN(C)OVAs with body mass; D, AN(C)OVAs with specimen-specific body size proxies; Phylogenetic signal. [file 12915_2021_1016_MOESM3_ESM.pdf]
