# Supplementary material for: Differing effects of size and lifestyle on bone structure in mammals
Source: BMC Biol. 2021 Apr 29;19:87. doi: 10.1186/s12915-021-01016-1 (PMC8086358; doi:10.1186/s12915-021-01016-1)
Supplement: Supplementary file 4 — Additional file 4. Phenograms depicting the reconstructed evolution of each trait among specialised clades. [file 12915_2021_1016_MOESM4_ESM.pdf]

Additional File 4 for:

Differing effects of size and lifestyle on bone structure in mammals

Eli Amson[1,*] & Faysal Bibi[1]

[1]Museum für Naturkunde, Leibniz-Institut für Evolutions- und Biodiversitätsforschung, Invalidenstraße 43, 10115 Berlin, Germany
*Corresponding author, eli.amson@mfn.berlin

Phenograms depicting the reconstructed evolution of each trait among specialised clades. For clade name abbreviations, see Supplementary File 2A. Time in millions of years. Trait are either size-corrected and/or without units. Function phenogram (phytools package; Revell 2012).

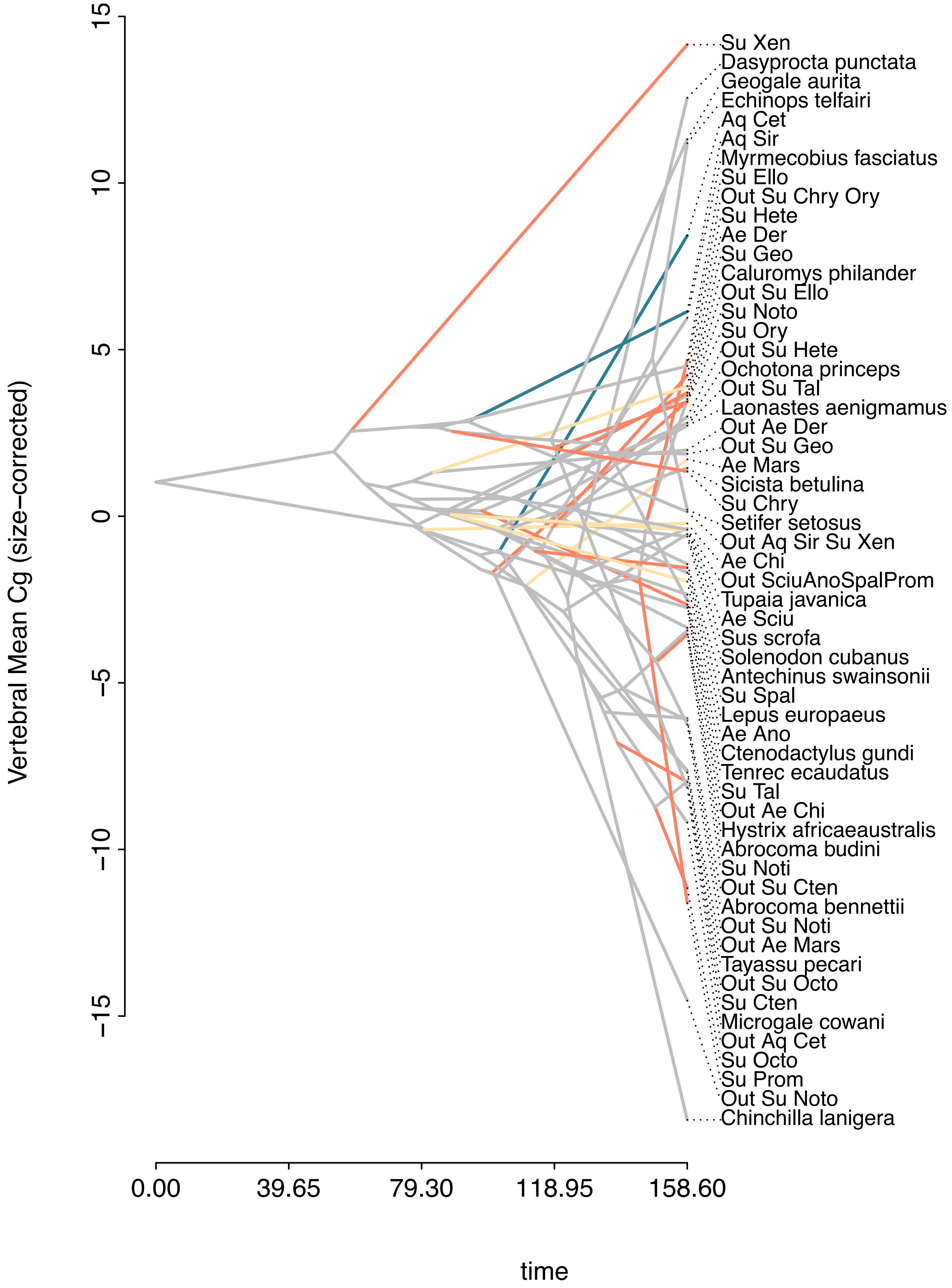

Vertebral Mean Cg (size−corrected)
15
10
5
0
−5
−10
−15
time
0.00
39.65
79.30
118.95
158.60
Su Xen
Dasyprocta punctata
Geogale aurita
Echinops telfairi
Aq Cet
Aq Sir
Myrmecobius fasciatus
Su Ello
Out Su Chry Ory
Su Hete
Ae Der
Su Geo
Caluromys philander
Out Su Ello
Su Noto
Su Ory
Out Su Hete
Ochotona princeps
Out Su Tal
Laonastes aenigmamus
Out Ae Der
Out Su Geo
Ae Mars
Sicista betulina
Su Chry
Setifer setosus
Out Aq Sir Su Xen
Ae Chi
Out SciuAnoSpalProm
Tupaia javanica
Ae Sciu
Sus scrofa
Solenodon cubanus
Antechinus swainsonii
Su Spal
Lepus europaeus
Ae Ano
Ctenodactylus gundi
Tenrec ecaudatus
Su Tal
Out Ae Chi
Hystrix africaeaustralis
Abrocoma budini
Su Noti
Out Su Cten
Abrocoma bennettii
Out Su Noti
Out Ae Mars
Tayassu pecari
Out Su Octo
Su Cten
Microgale cowani
Out Aq Cet
Su Octo
Su Prom
Out Su Noto
Chinchilla lanigera

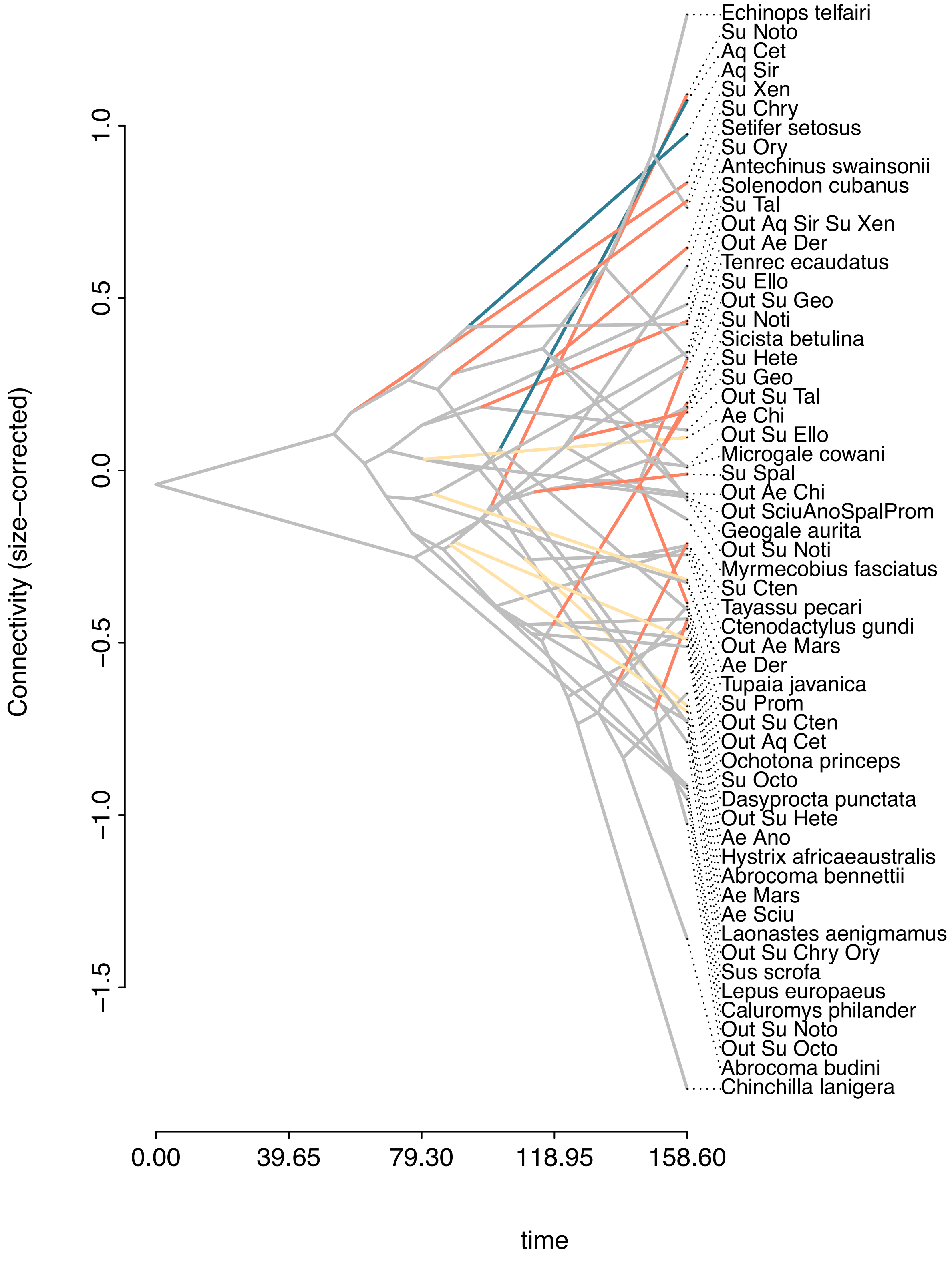

Connectivity (size–corrected)
1.0
0.5
0.0
−0.5
−1.0
−1.5
0.00
39.65
79.30
118.95
158.60
time
Echinops telfairi
Su Noto
Aq Cet
Aq Sir
Su Xen
Su Chry
Setifer setosus
Su Ory
Antechinus swainsonii
Solenodon cubanus
Su Tal
Out Aq Sir Su Xen
Out Ae Der
Tenrec ecaudatus
Su Ello
Out Su Geo
Su Noti
Sicista betulina
Su Hete
Su Geo
Out Su Tal
Ae Chi
Out Su Ello
Microgale cowani
Su Spal
Out Ae Chi
Out SciuAnoSpalProm
Geogale aurita
Out Su Noti
Myrmecobius fasciatus
Su Cten
Tayassu pecari
Ctenodactylus gundi
Out Ae Mars
Ae Der
Tupaia javanica
Su Prom
Out Su Cten
Out Aq Cet
Ochotona princeps
Su Octo
Dasyprocta punctata
Out Su Hete
Ae Ano
Hystrix africaeaustralis
Abrocoma bennettii
Ae Mars
Ae Sciu
Laonastes aenigmamus
Out Su Chry Ory
Sus scrofa
Lepus europaeus
Caluromys philander
Out Su Noto
Out Su Octo
Abrocoma budini
Chinchilla lanigera

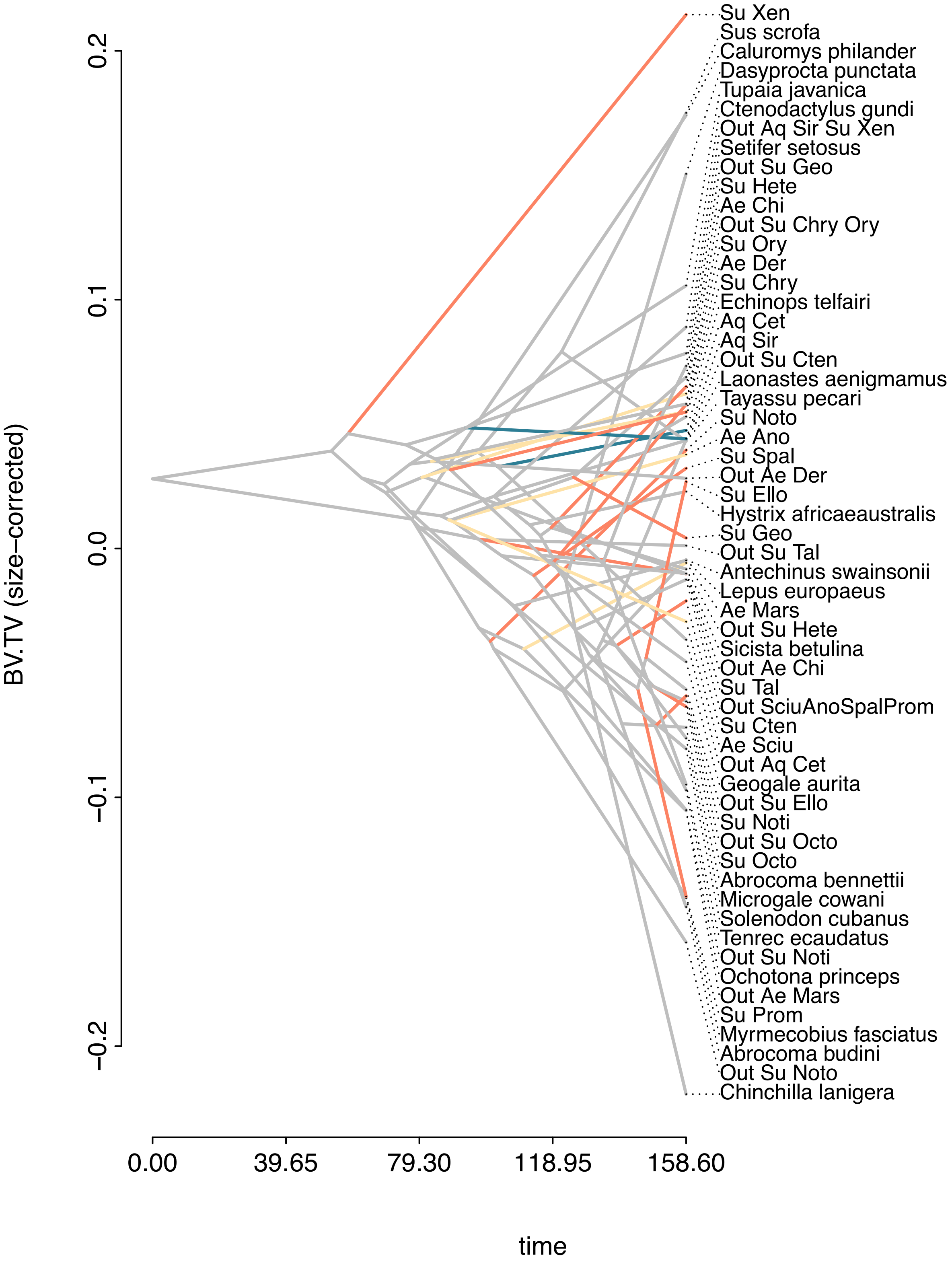

BV.TV (size−corrected)
0.2
0.1
0.0
−0.1
−0.2
0.00
39.65
79.30
118.95
158.60
time
Su Xen
Sus scrofa
Caluromys philander
Dasyprocta punctata
Tupaia javanica
Ctenodactylus gundi
Out Aq Sir Su Xen
Setifer setosus
Out Su Geo
Su Hete
Ae Chi
Out Su Chry Ory
Su Ory
Ae Der
Su Chry
Echinops telfairi
Aq Cet
Aq Sir
Out Su Cten
Laonastes aenigmamus
Tayassu pecari
Su Noto
Ae Ano
Su Spal
Out Ae Der
Su Ello
Hystrix africaeaustralis
Su Geo
Out Su Tal
Antechinus swainsonii
Lepus europaeus
Ae Mars
Out Su Hete
Sicista betulina
Out Ae Chi
Su Tal
Out SciuAnoSpalProm
Su Cten
Ae Sciu
Out Aq Cet
Geogale aurita
Out Su Ello
Su Noti
Out Su Octo
Su Octo
Abrocoma bennettii
Microgale cowani
Solenodon cubanus
Tenrec ecaudatus
Out Su Noti
Ochotona princeps
Out Ae Mars
Su Prom
Myrmecobius fasciatus
Abrocoma budini
Out Su Noto
Chinchilla lanigera

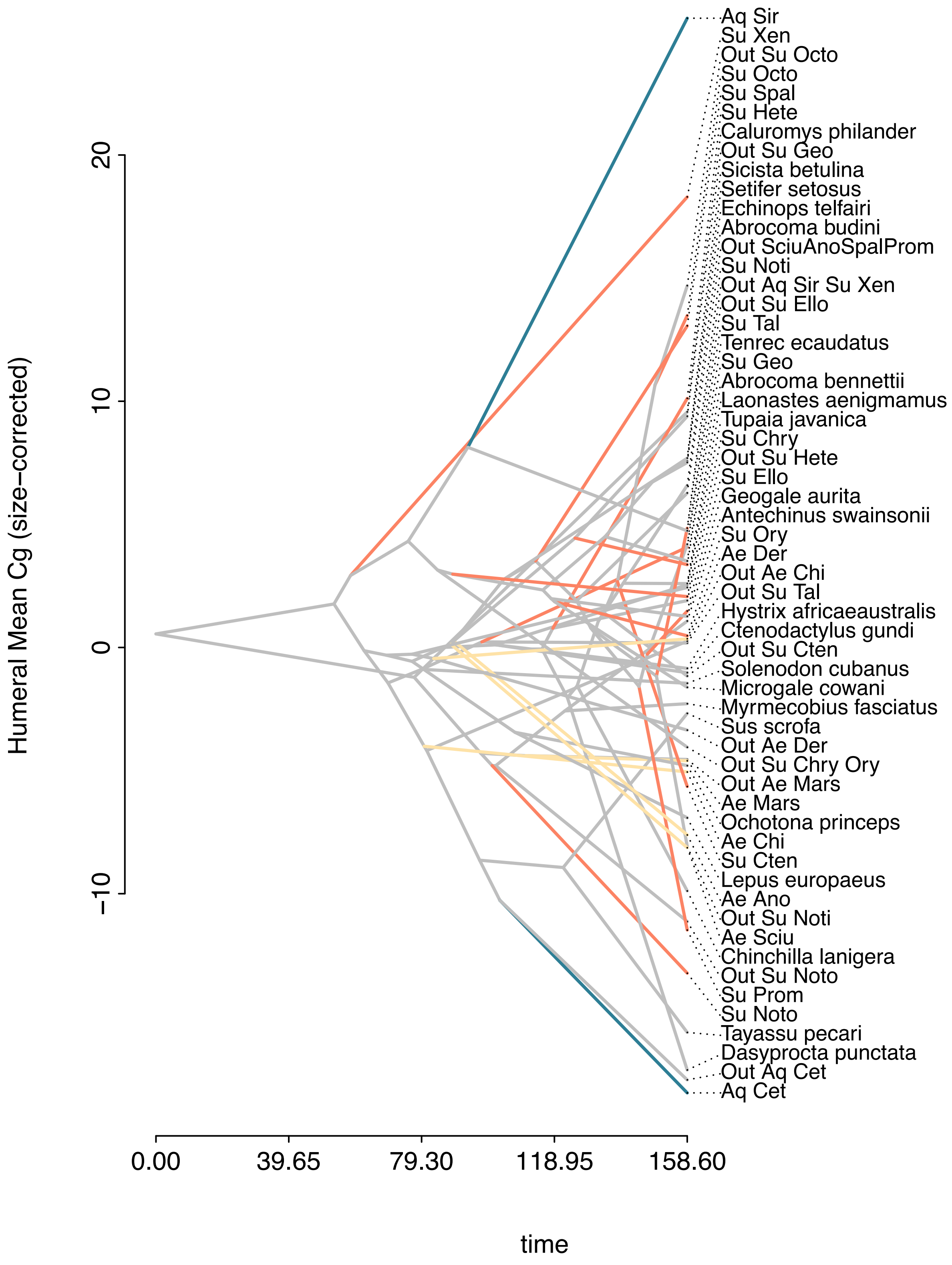

Humeral Mean Cg (size−corrected)
20
10
0
−10
0.00
39.65
79.30
118.95
158.60
time
Aq Sir
Su Xen
Out Su Octo
Su Octo
Su Spal
Su Hete
Caluromys philander
Out Su Geo
Sicista betulina
Setifer setosus
Echinops telfairi
Abrocoma budini
Out SciuAnoSpalProm
Su Noti
Out Aq Sir Su Xen
Out Su Ello
Su Tal
Tenrec ecaudatus
Su Geo
Abrocoma bennettii
Laonastes aenigmamus
Tupaia javanica
Su Chry
Out Su Hete
Su Ello
Geogale aurita
Antechinus swainsonii
Su Ory
Ae Der
Out Ae Chi
Out Su Tal
Hystrix africaeaustralis
Ctenodactylus gundi
Out Su Cten
Solenodon cubanus
Microgale cowani
Myrmecobius fasciatus
Sus scrofa
Out Ae Der
Out Su Chry Ory
Out Ae Mars
Ae Mars
Ochotona princeps
Ae Chi
Su Cten
Lepus europaeus
Ae Ano
Out Su Noti
Ae Sciu
Chinchilla lanigera
Out Su Noto
Su Prom
Su Noto
Tayassu pecari
Dasyprocta punctata
Out Aq Cet
Aq Cet

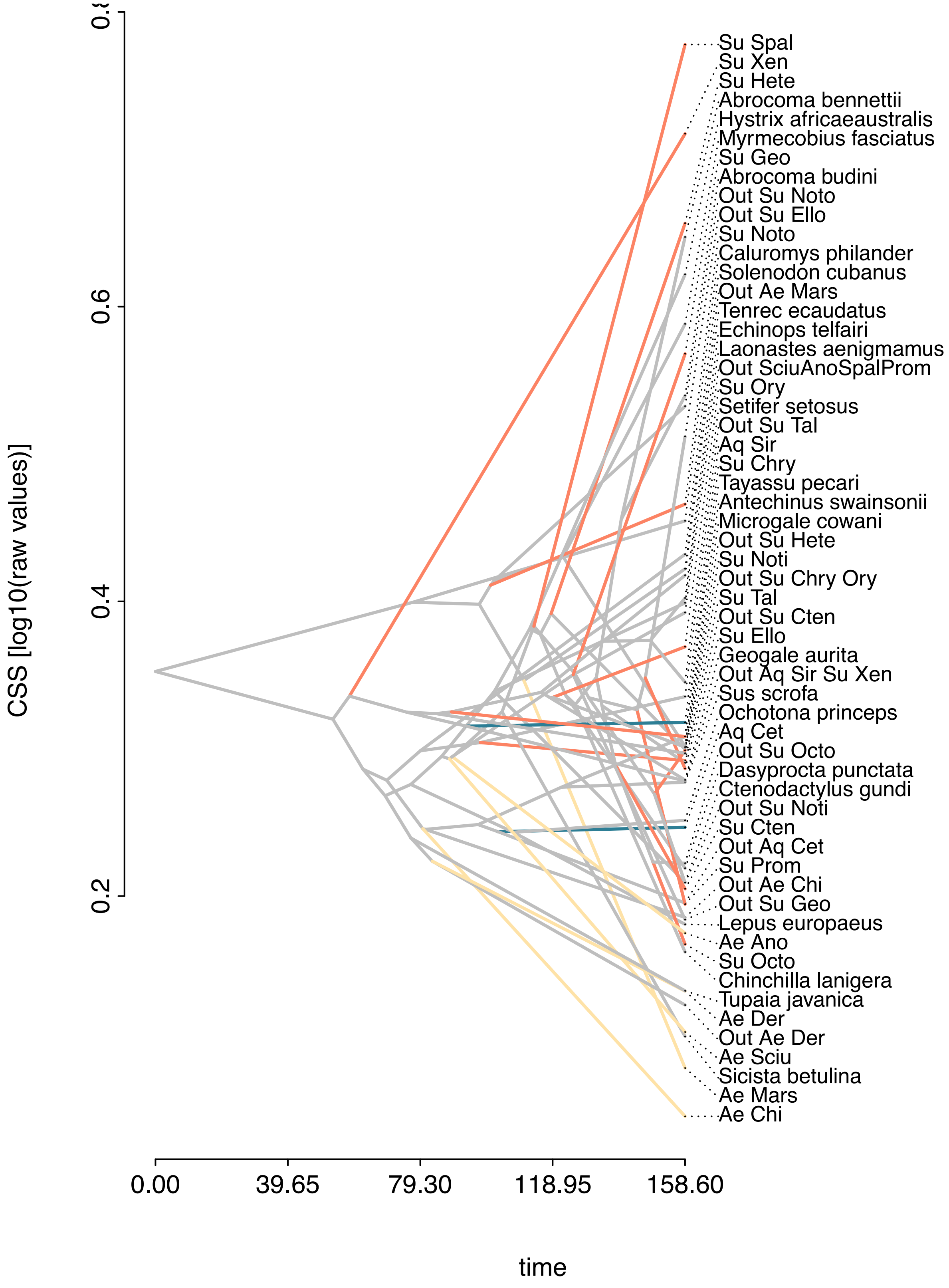
CSS [log10(raw values)]
0.2
0.4
0.6
0.8
time
0.00
39.65
79.30
118.95
158.60
Su Spal
Su Xen
Su Hete
Abrocoma bennettii
Hystrix africaeaustralis
Myrmecobius fasciatus
Su Geo
Abrocoma budini
Out Su Noto
Out Su Ello
Su Noto
Caluromys philander
Solenodon cubanus
Out Ae Mars
Tenrec ecaudatus
Echinops telfairi
Laonastes aenigmamus
Out SciuAnoSpalProm
Su Ory
Setifer setosus
Out Su Tal
Aq Sir
Su Chry
Tayassu pecari
Antechinus swainsonii
Microgale cowani
Out Su Hete
Su Noti
Out Su Chry Ory
Su Tal
Out Su Cten
Su Ello
Geogale aurita
Out Aq Sir Su Xen
Sus scrofa
Ochotona princeps
Aq Cet
Out Su Octo
Dasyprocta punctata
Ctenodactylus gundi
Out Su Noti
Su Cten
Out Aq Cet
Su Prom
Out Ae Chi
Out Su Geo
Lepus europaeus
Ae Ano
Su Octo
Chinchilla lanigera
Tupaia javanica
Ae Der
Out Ae Der
Ae Sciu
Sicista betulina
Ae Mars
Ae Chi

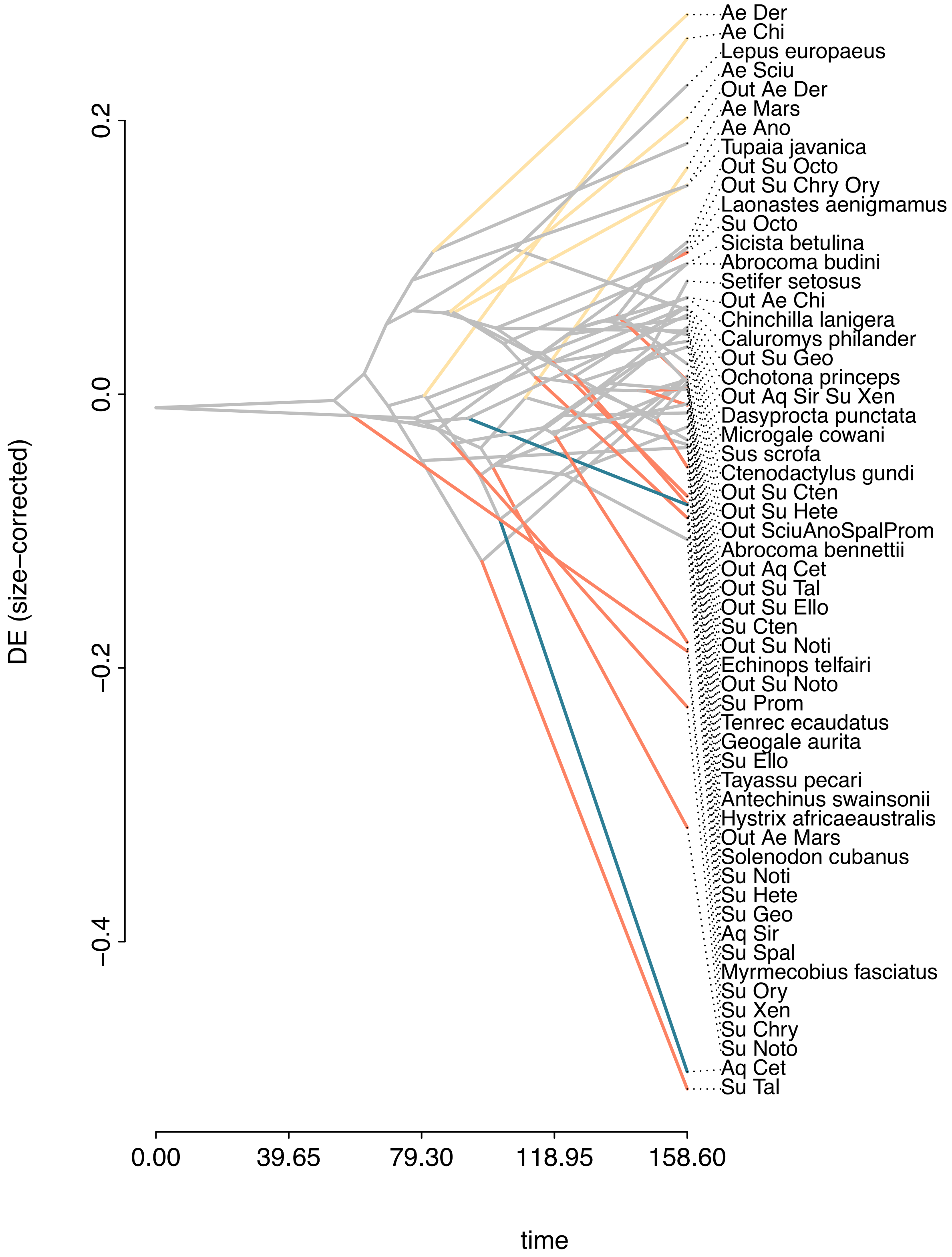
DE (size-corrected)
0.2
0.0
−0.2
−0.4
0.00
39.65
79.30
118.95
158.60
time
Ae Der
Ae Chi
Lepus europaeus
Ae Sciu
Out Ae Der
Ae Mars
Ae Ano
Tupaia javanica
Out Su Octo
Out Su Chry Ory
Laonastes aenigmamus
Su Octo
Sicista betulina
Abrocoma budini
Setifer setosus
Out Ae Chi
Chinchilla lanigera
Caluromys philander
Out Su Geo
Ochotona princeps
Out Aq Sir Su Xen
Dasyprocta punctata
Microgale cowani
Sus scrofa
Ctenodactylus gundi
Out Su Cten
Out Su Hete
Out SciuAnoSpalProm
Abrocoma bennettii
Out Aq Cet
Out Su Tal
Out Su Ello
Su Cten
Out Su Noti
Echinops telfairi
Out Su Noto
Su Prom
Tenrec ecaudatus
Geogale aurita
Su Ello
Tayassu pecari
Antechinus swainsonii
Hystrix africaeaustralis
Out Ae Mars
Solenodon cubanus
Su Noti
Su Hete
Su Geo
Aq Sir
Su Spal
Myrmecobius fasciatus
Su Ory
Su Xen
Su Chry
Su Noto
Aq Cet
Su Tal
