# Supplementary material for: Differing effects of size and lifestyle on bone structure in mammals
Source: BMC Biol. 2021 Apr 29;19:87. doi: 10.1186/s12915-021-01016-1 (PMC8086358; doi:10.1186/s12915-021-01016-1)

Additional File 4 for:

Differing effects of size and lifestyle on bone structure in mammals

Eli Amson[1,*] & Faysal Bibi[1]

[1]Museum für Naturkunde, Leibniz-Institut für Evolutions- und Biodiversitätsforschung, Invalidenstraße 43, 10115 Berlin, Germany
*Corresponding author, eli.amson@mfn.berlin

Additional trabecular parameters of the vertebral centrum acquired on a subset of the specimens for which the Connectivity exceeded 40 (as they are not meaningful for volumes of interest comprising few or no trabeculae). (**a**) Mean trabecular thickness; (**b**) Mean trabecular spacing; (**c**) degree of anisotropy; (**d**) mean direction of the trabeculae (MDT)

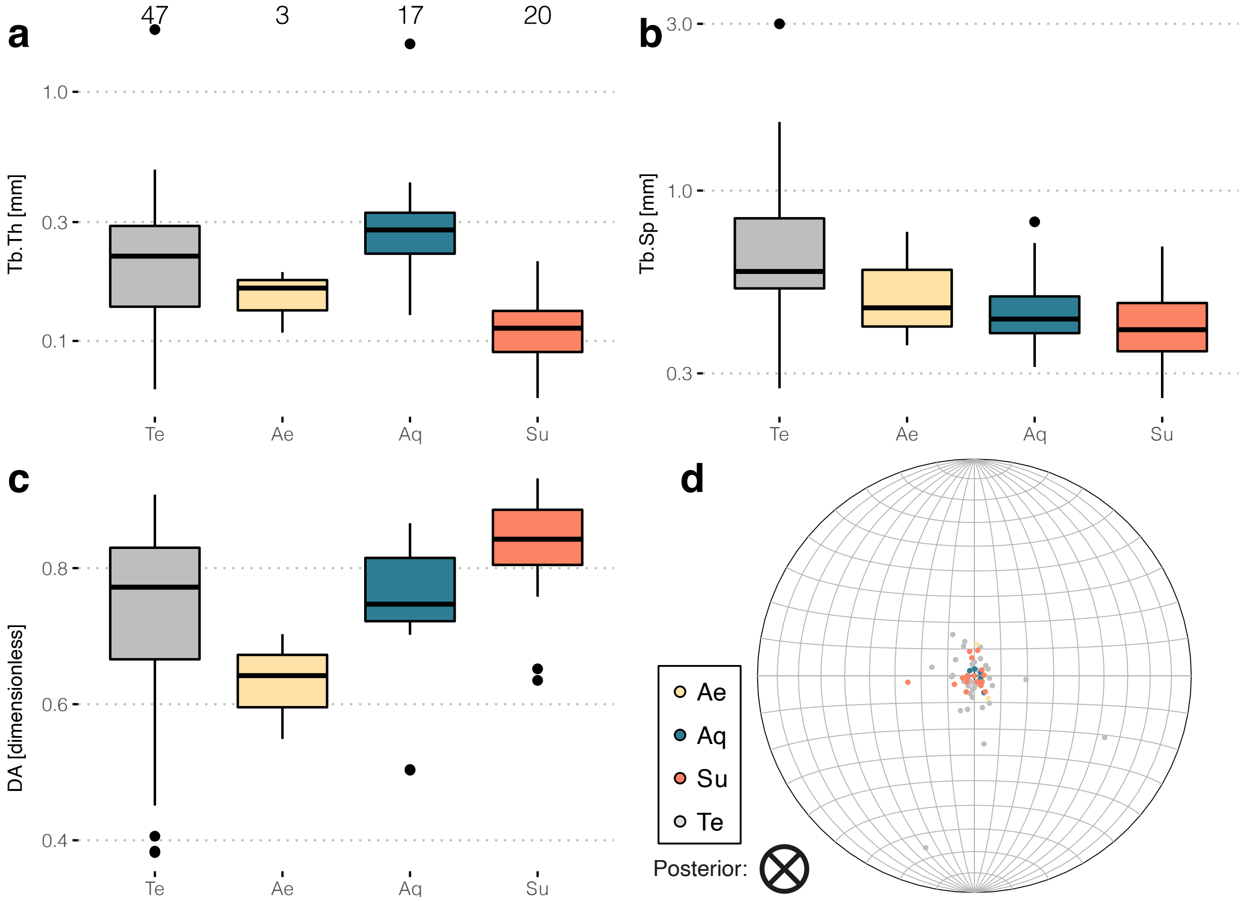

Supplement: Supplementary file 5 — Additional file 5. Additional trabecular parameters of the vertebral centrum: a, Trabecular Thickness (Tb.Th); b, Trabecular Spacing (Tb.Sp); c, Degree of Anisotropy (DA), and d, the main direction of the trabeculae (MDT). [file 12915_2021_1016_MOESM5_ESM.pdf]
